# Memristor Emulator Circuits: Recent Advances in Design Methodologies, Healthcare Applications, and Future Prospects

**DOI:** 10.3390/mi16070818

**Published:** 2025-07-17

**Authors:** Amel Neifar, Imen Barraj, Hassen Mestiri, Mohamed Masmoudi

**Affiliations:** 1 Systems Integration & Emerging Energies (SI2E), Electrical Engineering Department, National Engineering School of Sfax, University of Sfax, Sfax 3038, Tunisia; 2 Department of Computer Engineering, College of Computer Engineering and Sciences, Prince Sattam Bin Abdulaziz University, Al-Kharj 11942, Saudi Arabia

**Keywords:** memristor, memristor emulator, pinched hysteresis loop, CMOS, healthcare applications

## Abstract

Memristors, as the fourth fundamental circuit element, have attracted significant interest for their potential in analog signal processing, computing, and memory storage technologies. However, physical memristor implementations still face challenges in reproducibility, scalability, and integration with standard CMOS processes. Memristor emulator circuits, implemented using analog, digital, and mixed components, have emerged as practical alternatives, offering tunability, cost effectiveness, and compatibility with existing fabrication technologies for research and prototyping. This review paper provides a comprehensive analysis of recent advancements in memristor emulator design methodologies, including active and passive analog circuits, digital implementations, and hybrid approaches. A critical evaluation of these emulation techniques is conducted based on several performance metrics, including maximum operational frequency range, power consumption, and circuit topology. Additional parameters are also taken into account to ensure a comprehensive assessment. Furthermore, the paper examines promising healthcare applications of memristor and memristor emulators, focusing on their integration into biomedical systems. Finally, key challenges and promising directions for future research in memristor emulator development are outlined. Overall, the research presented highlights the promising future of memristor emulator technology in bridging the gap between theoretical memristor models and practical circuit implementations.

## 1. Introduction

The memristor, proposed by Leon Chua in 1971 as the fourth fundamental circuit element, completes the symmetry among charge, flux, voltage, and current in electrical networks [1]. Its eventual physical realization in 2008 by Hewlett-Packard Labs marked a major breakthrough, demonstrating a nanoscale device capable of non-volatile memory and synaptic-like plasticity [2]. Since then, memristors have been widely investigated for their potential to revolutionize the field of electronics and computing [3,4]. Despite their theoretical promise, practical memristor implementations face several practical challenges [5,6]. One of the most critical issues is fabrication inconsistency; even minor variations in material composition or manufacturing conditions can lead to significant deviations in memristive behavior. This unpredictability makes it difficult to mass-produce reliable devices, particularly for applications requiring high precision, such as analog neuromorphic circuits or memory arrays. In addition, integration with existing CMOS technology presents another major obstacle. Memristors often require specialized materials such as transition metal oxides and unconventional fabrication techniques that are incompatible with standard semiconductor processes [7]. This incompatibility limits their scalability and raises concerns about long-term reliability, especially in commercial electronics where CMOS dominance is well-established. As a result, researchers face a trade-off: either develop entirely new fabrication methods or find ways to adapt memristors to conventional silicon-based systems. Another concern is the limited operational frequency range of physical memristors. Many experimentally realized devices exhibit optimal performance only at low frequencies, restricting their use in high-speed applications such as RF systems or real-time signal processing [8]. Additionally, issues like cycle-to-cycle variability and degradation over time further complicate their deployment in practical circuits, where stability is non-negotiable [9]. These constraints have motivated research interest in memristor emulator circuits: electronic circuits that replicate memristive properties using conventional components. Emulators offer a flexible and cost-effective alternative, enabling researchers to explore memristor dynamics without requiring specialized fabrication processes. Furthermore, memristor emulator circuits can be easily integrated into existing electronic systems, allowing for seamless experimentation and testing. This approach has the potential to accelerate the development and implementation of memristor-based circuits in various applications.

Researchers have introduced various implementation techniques that balance complexity, performance, and practical considerations. Simplified designs using minimal components represent an important approach focused on accessibility and cost-effectiveness [10,11,12]. These compact circuits typically combine basic passive elements with a limited number of active devices. For applications that demand greater precision and adaptability, researchers have developed more advanced emulator architectures. These sophisticated designs integrate active analog components such as operational amplifiers, operational transconductance amplifiers (OTAs) [13,14], and second-generation current conveyors (CCIIs) [15,16] to enable dynamic tuning of the emulator’s behavior. This level of tunability is particularly advantageous in experimental settings, where flexible control over parameters is essential for validating theoretical models or exploring new computational paradigms. Moreover, it plays a critical role in the development of adaptive neuromorphic systems, where the ability to fine-tune synaptic weights and learning rules in hardware is key to achieving biologically inspired functionality and efficient learning mechanisms. The most advanced emulator implementations employ hybrid analog–digital architectures that combine circuit elements with programmable digital components [17]. Field-programmable gate arrays (FPGAs) and mixed-signal microcontrollers form the core of these designs, supporting features like real-time reconfiguration and adaptive learning algorithms [18,19]. Such hybrid approaches can closely mimic the complex dynamics of physical memristors while offering digital programmability. A distinct line of research has focused on MOSFET-only memristor emulator designs that minimize or entirely eliminate the use of passive and active components [20,21,22,23]. These implementations are specifically tailored for compatibility with standard CMOS fabrication processes, which enhances their suitability for seamless integration into conventional electronic systems. Dynamic threshold MOSFET (DTMOS) techniques have proven effective in enhancing circuit stability and reducing power consumption [24,25]. However, their limited frequency response due to factors like increased parasitic capacitance and slower switching speeds restricts their performance in high-frequency and real-time applications. Each of these design methodologies addresses different implementation priorities, reflecting the diverse requirements of memristor emulation across various application domains. The choice among simplicity, precision, programmability, or integration potential depends on the specific needs of the target system.

Memristor emulators have demonstrated remarkable versatility across multiple domains. In neuromorphic computing, they enable the prototyping of artificial synapses and spiking neural networks, facilitating energy-efficient brain-inspired architectures. Analog signal processing benefits from their nonlinear dynamics, where emulators are used in chaotic oscillators, filters, and adaptive amplifiers. Additionally, memory and logic circuits leverage emulators to study resistive switching behavior, aiding the development of next-generation non-volatile memory and reconfigurable computing systems. Beyond traditional electronics, emulators are increasingly employed in secure communications for chaos-based encryption, where their deterministic yet complex hysteresis enhances data protection. Among these applications, healthcare systems stand out as a particularly promising field for memristor emulators due to their compatibility with biomedical signal processing and neuromorphic diagnostics. By integrating emulators into biosignal processing circuits, researchers enhance the accuracy of EEG, ECG, and EMG analyses, leading to better early diagnosis of neurological and cardiovascular disorders. Furthermore, memristor emulators contribute advanced processing capabilities for medical imaging systems that improve image resolution in MRI and CT scans, enhance feature extraction, and accelerate reconstruction algorithms without compromising diagnostic quality. Thus, a systematic review of existing emulator topologies, their performance, and suitability for various applications is needed to guide future developments in this field.

The existing literature contains numerous comprehensive reviews examining memristor technologies, including their physical characteristics, material implementations [26,27,28], and diverse applications [4]. However, there is a noticeable gap in focused reviews specifically addressing memristor emulator circuits. In [29], the authors present a review discussing the research progress and applications of memristor emulator circuits. While the paper provides a broad overview, it lacks a comparative analysis, such as tabulated evaluations that would aid researchers in understanding the relative strengths and limitations of different emulator designs. Additionally, the classification scheme used to categorize emulator types is overly general and lacks specificity, limiting its practical utility. In another study [30], the authors focus exclusively on digital implementations of memristor-based emulators, particularly those realized through FPGA platforms, without addressing analog or hybrid approaches.

This review paper provides a comprehensive and systematic analysis of memristor emulator circuits, establishing a novel classification framework that distinguishes between five principal architectural approaches: CMOS, analog active, nonlinear passive emulators, and digital and hybrid implementations. Furthermore, existing emulator topologies are not only categorized but also critically examined in terms of their advantages, limitations, and optimal application domains. For each architectural class, specific subclasses have been defined, and key performance metrics such as maximum operating frequency, component type and count, circuit configuration (floating/grounded, incremental/decremental), and power consumption are evaluated. Additionally, innovative design techniques that extend the capabilities of current implementations are highlighted. Applications of memristor emulator circuits across various domains, particularly within healthcare systems, have also been reported. These include their integration into biomedical signal processing and medical image processing. Finally, the key challenges associated with current emulator technologies have been outlined, and potential future research directions have been presented to guide ongoing development in this field.

The remainder of this paper is structured as follows: Section 2 presents the fundamentals of memristor emulation, providing the theoretical background necessary to understand emulator behavior. Section 3 explores various memristor emulator circuit design approaches, offering a detailed classification and performance analysis. Section 4 discusses the applications of memristor emulators, with a focus on healthcare systems. Section 5 outlines the current challenges and future research directions in the field. Finally, Section 6 concludes the paper by summarizing key insights and contributions.

## 2. Fundamentals of Memristor Emulation

### 2.1. Memristor Theory

The ideal memristor was defined by Chua through a nonlinear relationship between charge (q) and magnetic flux (φ), completing the quartet of fundamental circuit variables, along with voltage (*v*) and current (*i*). This relationship can be expressed as [1]:

(1) φ=f(q)

where f(q) represents a continuous, differentiable function. Differentiating this equation with respect to time yields the voltage–current relationship that characterizes memristive behavior:

(2) v(t)=M(q(t))·i(t)

where M(q)=dφ/dq represents the memristance, a resistance that remembers its past. This memory property emerges because $q\left(t\right)=\int i(t)dt$, making the resistance dependent on the complete history of current flow [31]. Equation (3) illustrates this fundamental relationship.

(3) $$M\left(q\right)=R_{OFF}(1-\mu_{v}R_{ON}q(t)/D^{2})$$

where $R_{ON}$ and $R_{OFF}\;$ represent the minimum and maximum resistance states, $\mu_{v}\;$ is the average dopant mobility, D is the thickness of the active layer, and q(t) is the time-dependent charge passing through the device.

While the linear ion drift model effectively explains basic memristive behaviors like pinched hysteresis and state-dependent resistance, it has key limitations for emulator applications [32]. These include ignoring nonlinear boundary effects near electrodes, assuming constant dopant mobility despite field-dependent variations, and failing to capture frequency-dependent phenomena like hysteresis collapse. These issues arise from the model’s oversimplified representation of the physical processes behind memristive switching. To address these issues, researchers have developed more sophisticated models that better represent real device behavior. The nonlinear drift model introduces a windowing function to account for boundary effects, while the Simmons tunnel barrier model provides a more accurate description of conduction mechanisms. For high-frequency operation where nonlinear ion drift effects become dominant, the Simmons tunnel barrier model [33] offers a more physically accurate representation. However, the implementation of this model in emulator circuits presents significant challenges due to the computational complexity of the exponential relationship and the need for precise parameter tuning to match specific device characteristics. The Biolek window function model [34] represents an important step in this direction by modifying the linear drift framework with additional state-dependent terms:

(4) $$M\left(q\right)=R_{OFF}-\left(R_{OFF}-R_{ON}\right)\;x(t)$$

(5) $$\frac{dx}{dt}=k\;i\left(t\right)\;f(x)$$

where x(t) is a normalized state variable (0≤x(t) ≤ 1), k is a scaling factor, and f(x) is a window function that prevents unphysical boundary conditions. This model offers several advantages for emulator implementation: it maintains computational efficiency, avoids the infinite resistance states in simpler models, and provides better agreement with experimental observations of device behavior near the boundaries. The window function approach has proven particularly valuable for emulating the gradual resistance changes observed in neuromorphic applications, where precise control over state transitions is essential.

The previously discussed models provide physically grounded descriptions of memristive behavior by modeling underlying mechanisms like ion migration and quantum tunneling. While these approaches offer valuable insights into device physics, their computational complexity and parameter sensitivity often make them impractical for large-scale circuit simulations. This limitation motivated the development of simplified, behavior-based models that capture essential memristive characteristics while maintaining computational efficiency. The TEAM (threshold adaptive memristor) and VTEAM (voltage-controlled TEAM) models represent this approach [35]. The TEAM model introduces a threshold-based approach that simplifies computational requirements. The model ensures that no state change occurs unless the current crosses the threshold values, making it particularly suitable for digital circuit simulations. The VTEAM model extends the threshold concept to voltage-controlled operations while maintaining computational efficiency [36]:

(6) $$\frac{dw\left(t\right)}{dt}=\left\{\begin{array}{c} k_{off}\;{\left(\left(\frac{v\left(t\right)}{v_{off}}\right)-1\right)}^{\alpha_{off}}\;f_{off}\left(w\right)\;;\;0<v_{off}<v\;\\ \;0\;;\;v_{on}<v<v_{off}\\ k_{on}\;{\left(\left(\frac{v\left(t\right)}{v_{on}}\right)-1\right)}^{\alpha_{on}}\;f_{on}\left(w\right)\;v<v_{on}<0\; \end{array}\right.$$

The transition to voltage control offers significant advantages for modern memristor applications. Many emerging resistive memory technologies, particularly oxide-based RRAM devices, exhibit voltage-dependent switching that is more naturally described by VTEAM. The model maintains TEAM’s computational efficiency while better representing the physical operation of these devices. VTEAM’s voltage thresholds also align more closely with practical circuit design considerations, where voltage signals are typically easier to control precisely than currents. Despite these improvements, VTEAM shares several limitations. The model still relies on empirical parameter fitting rather than physical first principles, potentially limiting its predictive capability for novel device structures.

Table 1 presents a systematic comparison of the memristor models discussed. This comparison reveals several important considerations for emulator development. The comprehensive analysis of memristor models from physically grounded approaches like the linear/nonlinear ion drift and Simmons tunnel barrier models to the computationally efficient TEAM and VTEAM formulations reveals a fundamental trade-off between physical accuracy and practical implementation. While these mathematical models serve as valuable tools for simulation and theoretical understanding, they also highlight the need for practical hardware implementations that can replicate memristive behavior in real-world circuits. This transition from software modeling to hardware emulation presents significant opportunities for researchers and engineers to develop physical memristor emulators that can be experimentally validated and integrated into electronic systems.

Furthermore, while linear drift models provide computational simplicity and ease of implementation, their idealized assumptions often limit their accuracy in analog applications, where precise switching behavior is critical. In contrast, nonlinear or physics-based models such as VTEAM capture more realistic memristive dynamics, including threshold effects and nonlinear ion drift, at the expense of increased computational overhead. For memory and storage emulation, TEAM/VTEAM models are particularly advantageous due to their configurable thresholds, enabling precise control over state transitions. Meanwhile, neuromorphic circuit design benefits from nonlinear or stochastic models, which more faithfully replicate the gradual conductance changes and variability inherent in biological synaptic plasticity.

### 2.2. Key Emulator Characteristics

Memristor emulators aim to replicate the essential behaviors of physical memristive devices using conventional electronic components. Among the most critical characteristics that define an effective emulator are the pinched hysteresis loop, frequency dependence, and nonvolatility characteristics. These properties determine how closely the emulator mimics real memristors in dynamic operation, switching speed, and state retention. Below, we examine the characteristics in detail, discussing their importance and implementation challenges in emulator circuits.

#### 2.2.1. Pinched Hysteresis Loop

**Figure 1:**
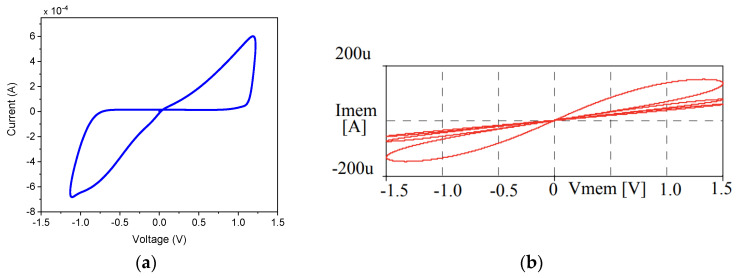
(**a**) Memristor pinched hysteresis loop [12]. (**b**) Frequency dependence of the pinched hysteresis loop, showing how the loop narrows as frequency increases [34].

The pinched hysteresis loop serves as the definitive fingerprint of memristive behavior, providing critical insights into the quality and accuracy of memristor emulators. This distinctive pattern in the voltage–current characteristic arises from the fundamental property that the memristor’s resistance depends on its state history. When driven by a sinusoidal voltage $v(t)=V_{0}sin(\omega t)$, the current response i(t) becomes modulated by the time-varying memristance M(x(t)), creating the characteristic hysteresis lobes, as depicted in Figure 1a. The shape and evolution of these lobes with frequency reveal essential information about the emulator’s dynamics and fidelity to ideal memristor behavior. For emulators, replicating this loop with high fidelity is critical, as it directly impacts their ability to mimic real memristive devices in applications ranging from neuromorphic computing to chaotic systems.

Quantitative analysis of the hysteresis loop focuses on three key metrics: pinch point location, lobe area scaling, and slope symmetry. An ideal emulator maintains the pinch point precisely at the origin (0,0), with practical implementations achieving offsets < 1% of full scale. Practical emulator implementations face several challenges in reproducing ideal hysteresis characteristics. Component nonlinearities such as op-amp saturation and MOSFET threshold effects can distort lobe shapes, while parasitic capacitances introduce artificial phase shifts that inflate hysteresis areas. The region near the pinch point proves particularly sensitive to noise due to the theoretically infinite di/dv slope at this operating point. Various measurement approaches have been developed to characterize these effects, ranging from simple analog oscilloscope XY-mode measurements to precision impedance analyzer techniques.

#### 2.2.2. Frequency Dependence

The frequency response of memristor emulators represents a critical performance parameter that directly impacts their suitability for different applications. As the driving signal frequency increases, the emulator’s ability to faithfully reproduce memristive characteristics undergoes predictable but technologically challenging transformations. The transition from memory-dominated to resistive behavior follows a well-defined cutoff frequency ($f_{C}$), which represents the maximum operating frequency. This cutoff marks the point where the state variable can no longer follow the rapid oscillations of the input signal, causing the distinctive pinched hysteresis loop to gradually collapse into a linear resistance characteristic, as depicted in Figure 1b. Two distinct frequency regimes emerge in memristor emulator operation. At frequencies lower than the critical frequency (${f\ll f}_{C}$), ideal memristive behavior is exhibited, characterized by fully developed hysteresis lobes. As the frequency increases, a gradual narrowing of the hysteresis width is observed. When the frequency exceeds the maximum operating frequency ${(f\gg f}_{C}$), the emulator transitions into a purely resistive state, and its memristive properties are effectively lost. The frequency limitations of emulator circuits stem from several physical constraints. Active components such as operational amplifiers exhibit finite-gain bandwidth products that restrict high-frequency operations, typically limiting practical emulators to a few megahertz in analog implementations. Parasitic capacitances in discrete component implementations create unwanted low-pass filtering effects, while digital emulators face sampling rate limitations in their state variable updates. Modern design approaches combat these limitations through techniques such as frequency compensation networks, current-mode circuits that inherently operate at higher speeds, and hybrid analog–digital architectures that combine the bandwidth of analog front-ends with the precision of digital state variable control.

#### 2.2.3. Nonvolatility Characteristics

Nonvolatility is a defining characteristic of memristor emulators, enabling them to retain their resistive state even after the removal of the external power supply. This property stems from the emulator’s ability to mimic the ion drift and redistribution mechanisms observed in physical memristive devices, where the resistance state is determined by the spatial configuration of dopants or defects within the active layer. Unlike volatile memory elements, which lose their state upon power interruption, a well-designed memristor emulator preserves its resistance value due to the nonvolatile nature of the underlying switching mechanism. This feature is crucial for applications such as neuromorphic computing, analog memory, and energy-efficient edge devices, where state retention is essential for reliable operation. The degree of nonvolatility depends on factors such as material properties, interfacial effects, and the stability of the programmed state against thermal and temporal relaxation. By faithfully replicating this behavior, memristor emulators serve as valuable tools for prototyping and studying nonvolatile memory systems without requiring physical memristive devices.

## 3. Memristor Emulator Circuit Design Approaches

The field of memristor emulator circuits is highly varied, and many classification methods have been introduced in the literature to organize their different designs and functions. These include categorizations based on operating principles, where emulators are differentiated by their control mechanisms, such as voltage-controlled (memristance modulated by applied voltage) or current-controlled (state changes governed by charge flux) architectures. Other classifications emphasize application scope, distinguishing emulators tailored for neuromorphic computing, memory/logic circuits, or analog signal processing. Additional frameworks focus on programmability, segregating designs with fixed parameters (static memristive behavior) from tunable emulators (adjustable via external voltages, digital interfaces, or software), and port configuration, which contrasts floating emulators (requiring isolated power supplies) with grounded designs (simplified for integration into common ground-referenced systems). Each framework presents distinct design characteristics.

For this review, we adopt circuit implementation methodology as the primary classification criterion, a choice motivated by its ability to dissect the structural and technological distinctions that fundamentally shape emulator performance and applicability. As shown in Table 2, the existing memristor emulator circuits in the literature can be systematically categorized into four distinct classes:

- CMOS-based emulators: These designs utilize standard CMOS technology to replicate memristive behavior through carefully configured transistor networks, offering the advantages of scalability and compatibility with existing integrated circuit processes.
- Analog active emulators: This class employs active circuit elements such as operational amplifiers, multipliers, and analog computational blocks to dynamically generate the characteristic pinched hysteresis loops of memristors.
- Nonlinear passive emulators: Implemented using only passive components with inherent nonlinear characteristics, these emulators achieve memristive-like responses through combinations of resistors, capacitors, and diodes.
- Digital emulators: Use microcontroller units or FPGAs to numerically model memristive dynamics
- Hybrid emulators: Combining digital signal processing with analog interfaces while maintaining electrical compatibility with analog systems.

This comprehensive classification framework enables researchers to effectively compare design trade-offs between different implementation approaches, considering factors such as power consumption, maximum operating frequency response, and hardware complexity. The proposed categorization also highlights the diverse strategies employed to overcome the challenges of memristor emulation while providing guidance for selecting appropriate architectures based on specific application requirements.

**Table 2:** Classification of memristor emulator by circuit implementation methodology.

Type	Subclass	Key Characteristics	References Example
CMOS-Based	CMOS–Transistor-Based	Uses MOSFETs to emulate memristance; compact, low-power, compatible with IC processes	[13,21,23,37,38,39,40,41,42,43,44,45,46,47,48]
	DTMOS-Based	Dynamic threshold MOSFETs for better nonlinearity, improved tunability, low-voltage operation	[11,12,20,22,24,25]
Active analog	OTA/Op-Amp-Based	Precise hysteresis control	[13,14,49,50,51,52,53,54]
	Current-Mode (CFOA/CCII)	High-frequency operation and reduced parasitics	[15,16,55,56,57,58,59,60,61]
	Differential (DVCC/DXCCII)	Floating/grounded designs, symmetric hysteresis, and better noise immunity	[62,63,64,65,66,67,68,69,70,71,72,73,74,75,76]
	Mixed	Combines voltage/current-mode techniques for wider dynamic range	[77,78,79,80,81,82,83,84,85,86,87,88,89,90,91,92]
Nonlinear/Passive	Passive Networks	Diode/RLC-based designs for no-power applications	[79,93,94,95,96,97,98,99,100,101,102,103,104]
Digital	FPGA-Based	Programmable logic for real-time parameter adjustment and high flexibility	[18,19,105,106,107,108]
	Microcontroller-Based	Arduino/Raspberry Pi-driven emulators	[109,110,111,112,113,114]
Hybrid	Analog Core + Digital Tuning	Analog emulator with digital calibration for stability	[17,115,116]

### 3.1. CMOS-Based Emulators

CMOS-based emulators offer significant advantages due to their full compatibility with standard fabrication processes, enabling scalable and cost-effective integration into conventional electronic systems. Their inherent low-power operation, combined with good scalability, makes them particularly attractive for emerging applications such as neuromorphic computing, analog signal processing, and reconfigurable electronics. By utilizing MOSFETs in various configurations, such as differential pairs, cascode structures, or dynamic threshold (DTMOS) designs, CMOS-based emulators can mimic key memristive properties, including pinched hysteresis, frequency-dependent resistance modulation, and nonvolatile state retention. Their design flexibility allows for both floating and grounded topologies, satisfying diverse circuit requirements. Recent advances have further enhanced their performance through subthreshold operation, improved linearity, and hybrid digital tuning. In this section, we review the evolution of CMOS-based memristor emulators, analyzing their design methodologies, performance trade-offs, and emerging trends in the field.

**Figure 2:**
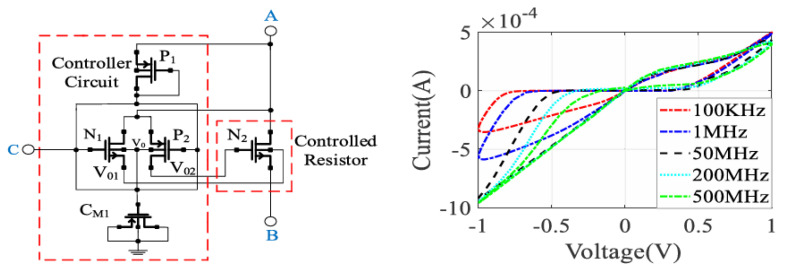
Proposed MRE design utilizing MOSCAP and corresponding pinched hysteresis loop at different input frequencies [11].

A novel CMOS-based memristor emulator (MRE) design utilizing a dynamic threshold MOS technique for high-frequency operation is introduced in [11]. As depicted in Figure 2, the proposed circuit consists of a controller circuit and a controlled resistor, implemented using four MOSFETs (two nMOS and two pMOS) and a capacitor (C1). The design eliminates the need for external biasing, resulting in zero static power consumption, while leveraging the dynamic threshold technique to enhance performance in low-power and low-voltage applications. Additionally, a comprehensive analysis of the equations governing the behavior of the MRE is presented. It explores the dynamics of voltage control, the memductance as a function of control voltages, parasitic influences, and process parameters. Furthermore, the study derives the unity–gain bandwidth, demonstrating its dependence on transconductance and parasitic capacitances. A key observation is that the memristance increases with applied pulse trains, exhibiting incremental behavior. The hysteresis loop’s shape is frequency-dependent, collapsing at high frequencies where the MRE behaves like a time-independent resistor. The design is validated using Cadence Virtuoso with a 180 nm CMOS process and tested experimentally with MOSFET arrays. Key findings include stable hysteresis loop behavior despite parasitic effects, reliable operation across the frequency range up to 500 MHz and temperature range from −30 °C to 100 °C, and optimized noise performance. The device demonstrates resilience across various process corners and supports resistive, capacitive, and inductive loads, enhancing its versatility. Post-layout simulations show minimal deviation from pre-layout results, while Monte Carlo analysis confirms robustness against process variations. Experimental validation further establishes the presence of a pinched hysteresis loop at frequencies up to 25 MHz, reinforcing the design’s reliability and efficiency. Therefore, this MRE presents a practical, low-power, and high-frequency CMOS memristor emulator with robust performance across process and temperature variations. The mathematical framework and experimental validation demonstrate its suitability for neuromorphic computing, analog memory, and signal processing applications. Future work could explore scaling to advanced nodes (e.g., FinFETs) for higher frequency and lower parasitics.

**Figure 3:**
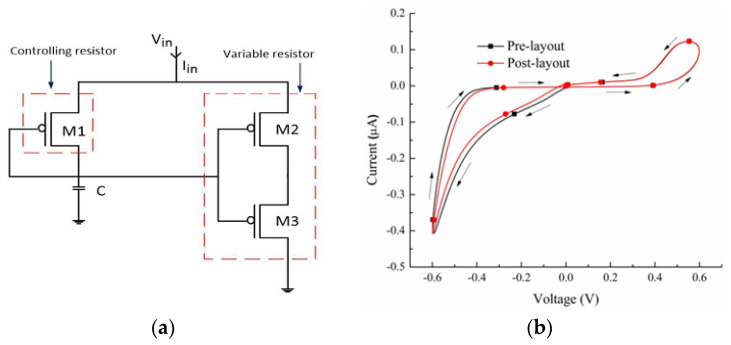
Proposed memristor model in [37] (**a**) CMOS circuit and (**b**) pre-layout vs. post-layout results.

A grounded memristor emulator composed of a controller circuit (PMOS M1 and capacitor C) and a variable resistor (PMOS M2 and M3) is introduced in [37]. The design leverages PMOS transistors for their low threshold variation and noise immunity. Figure 3 illustrates the MRE circuit. M1 operates in saturation, controlling the capacitor voltage ($V_{C}$), which drives the gates of M2 and M3 (operating in linear region) to produce memristive behavior. The mathematical model derives the memductance (W(ϕ)) as a function of flux, confirming flux-controlled dynamics. The emulator exhibits pinched hysteresis loops, a characteristic of memristive systems, though minor asymmetry arises due to parasitic effects. At high frequencies, hysteresis diminishes, transitioning to resistive behavior, aligning with the ideal memristor model. The design is highly energy-efficient, requiring zero static power and consuming only 175 nW dynamically. Validation through simulations in 90 nm CMOS and experimental testing with CD4007 MOS ICs confirms its functionality. The emulator demonstrates non-volatile memory, with incremental resistance changes persisting without power, mimicking synaptic plasticity. Process corner analysis shows stable performance, with a R_OFF_/R_ON_ ratio of approximately 59, comparable to TiO_2_-based memristors. Additionally, the design maintains minimal input noise and a compact layout with negligible pre/post-layout deviation. Experimental feasibility is demonstrated through off-the-shelf tests at 24 MHz with a 15 pF capacitor, confirming real-world applicability. These findings collectively establish the emulator’s robustness, efficiency, and potential for advanced electronic integration.

A two MOS transistor-based floating memristor circuit is introduced in [13]. The circuit utilizes a feedback mechanism where M1 (NMOS) controls the gate voltage of M2 (PMOS), which operates in the linear region to emulate memristance. Stability is maintained by a current source (CS) and supply voltage. Parasitic capacitance is leveraged to eliminate the need for external capacitors, reducing circuit complexity. Memductance is mathematically derived, showing strong hysteresis at low frequencies and a transition to resistive behavior at high frequencies. Performance validation confirms stable operation from 380 KHz to 1 GHz and temperature resilience from −40 °C to +85 °C. The circuit exhibits non-volatility, retaining its state without power, and remains robust across process variations. Monte Carlo analysis indicates less than 10% current deviation under parameter variations. Post-layout results closely align with pre-layout simulations, with minor hysteresis area reduction due to added parasitic resistance and capacitance. These findings highlight the circuit’s efficiency, stability, and adaptability. Another floating MRE using four MOSFETs (including a DTMOS transistor) and an external capacitor is introduced in [24]. The circuit dynamically adjusts threshold voltage based on input signals, enhancing speed and reducing leakage. It consists of a tunable resistor (M1 & M2) and a feedback control mechanism (M3 & M4), with a capacitor ensuring non-volatility. Memductance combines a fixed resistance with a flux-dependent memristance, exhibiting strong hysteresis at low frequencies and transitioning to resistive behavior at high frequencies. Simulations using the TSMC 180 nm PDK confirm an adjustable frequency range from DC to 3 MHz, with a pinched hysteresis loop retaining its key fingerprint. The circuit demonstrates non-volatility and remains stable across process corners (FF, TT, SS) and temperatures (−40 °C to +85 °C). Monte Carlo analysis indicates minimal current deviation (<10%). Experimental tests using ALD1106 MOSFETs validate frequency scalability from 500 Hz to 500 KHz, with the hysteresis shrinking at higher frequencies. The design is compact (157.48 µm^2^) and energy-efficient, consuming only 8.24 µW.

The authors in [20] have introduced a high-frequency, compact, MOS-only memristor emulator (PGME/NGME) that eliminates external capacitors and power supplies, relying solely on transistor intrinsic capacitances to achieve memory effects. With GHz-level operation reaching up to 5 GHz and an ultra-compact chip area, the design is well-suited for high-speed neuromorphic and logic-in-memory applications. The emulator features a transistor-only architecture, where the PGME (P-type) employs four PMOS transistors, while the NGME (N-type) has four NMOS transistors. Memory effects are entirely driven by MOSFET gate, drain, and source capacitances, eliminating the need for external components. Ultra-high-frequency operation is enabled by intrinsic capacitances, allowing faster state switching compared to conventional RC-based emulators. The design supports Material Implication (IMPLY) logic, achieving an impressive 70 ps step time for high-speed Boolean operations. Additionally, the emulator maintains an extremely small area, with PGME-3 measuring only 3.9 × 2.7 μm^2^. Experimental validation includes testing with a discrete PMOS (CD4007 IC) in a chaotic oscillator circuit and simulations using TSMC 65 nm technology, both confirming GHz-level performance. The applications and advantages of the design make it highly effective for high-speed computing tasks, particularly in logic-in-memory operations and neuromorphic acceleration. By eliminating bulky capacitors, the emulator enables dense integration while maintaining zero static power consumption, significantly enhancing energy efficiency.

Furthermore, a floating memristor emulator using three nMOS transistors and one MOS capacitor, designed for high-frequency signal processing, is presented in [38]. The key innovation lies in the body-to-drain connection of nMOS devices, enhancing nonlinearity and enabling memristive behavior without external biasing. The circuit employs three nMOS transistors with body-drain connections to introduce nonlinearity and an MOS capacitor for charge storage, ensuring non-volatility. Its floating configuration eliminates ground dependency, allowing flexible integration. Memductance is expressed in terms of transconductance parameters and flux, showing strong pinched hysteresis loops at low frequencies while transitioning to linear resistor behavior at higher frequencies. Simulations in a 90 nm CMOS process confirm frequency scalability from 50 KHz to 10 MHz, with robust process and temperature stability (−40 °C to +85 °C). The circuit retains its state during pulse “OFF” periods, confirming non-volatility. Experimental tests using IRF840 MOSFETs validate frequency scalability from 100 KHz to 6 MHz with a 22 nF capacitor, while post-layout simulations closely match schematic predictions. With an ultra-compact layout of 59.41 µm^2^, the design is highly suited for VLSI applications, offering efficiency, stability, and adaptability. The authors in [39] have proposed a compact and efficient memristor emulator circuit implemented using only four MOS transistors in a TSMC 180 nm CMOS process. The design leverages pMOS transistors (M1–M4) with a capacitive coupling mechanism to control the gate voltage of M3 (V_G_ = V_C_ = −g_m12_I_N_/C). The derived memductance equation consists of two key components: a time-invariant part dependent on the threshold voltage (V_TP_) of M3 and a time-variant part governed by the input flux (I_N_). This structure enables the circuit to exhibit incremental memristive behavior, where the bulk terminal of M3 can be independently biased to tune the linear memductance, provided the source-bulk diode remains reverse-biased. Frequency analysis reveals the circuit’s operational limits at low frequencies (10–100 MHz), displaying a clear pinched hysteresis loop, while at higher frequencies, the time-variant component diminishes, reducing the emulator to a linear resistor. The circuit’s robustness is demonstrated through extensive simulations, including Monte Carlo analyses, which confirm stable operation across process corners (SS, TT, FF) and temperature variations (−40 °C to 80 °C), with slight deviations in hysteresis loop size. Experimental validation using discrete ALD1116/ALD1117 transistors and a 100 nF capacitor successfully emulates the behavior of a fabricated ZnO memristor at ultralow frequencies (4–20 Hz), showcasing the design’s versatility. The emulator’s advantages include its minimal transistor count, high-frequency capability (up to 100 MHz in simulations), and low-voltage operation (±0.9 V), making it suitable for integration in analog neuromorphic systems. However, practical high-frequency operation requires femto-farad capacitors, posing a challenge for breadboard implementations.

The simplest implementation comes from the MOS-only memristor emulator using just two MOSFETs without external components [23]. This design leverages intrinsic transistor capacitances to achieve operation up to 300 MHz while maintaining an ultra-compact 1.8 × 2.57 μm^2^ layout area in 65 nm CMOS. Building on this minimalist approach, another study [22] introduces DTMOS-based emulators that completely eliminate DC bias requirements. These floating and grounded configurations demonstrate zero static power consumption while maintaining full functionality, verified through both simulations using 180 nm CMOS technology and experimental tests with ALD1116/ALD1117 MOSFETs. For applications requiring more precise control, a single-MOSFET design incorporating an RC filter [25] offers an interesting compromise. With a slightly smaller layout area (1.586 μm^2^) and limited to 80 MHz operation, this passive emulator achieves remarkable pW-range dynamic power consumption and has been experimentally validated using ALD1117 MOSFETs. The inclusion of the RC filter provides stable operation while maintaining the benefits of minimal active components.

The most advanced implementation [40] pushes the boundaries of memristor emulation with a 2T1C (two-transistor, one-capacitor) design that achieves 150 MHz operation at just 13.1 μW power consumption in a 1.25 μm^2^ area. Notably, this work introduces the first cryogenic CMOS memristor emulator (Cryo-2T1C) capable of operation at 4.2 K, with low power consumption (47 μW) and area (3.1 μm^2^). Both room-temperature and cryogenic variants demonstrate robust performance across process variations, making them particularly suitable for emerging computing systems.

In this section, we have analyzed several published CMOS-based memristor emulator circuits, highlighting their key design approaches and performance characteristics. However, the existing literature includes additional works that employ alternative topologies or optimization techniques beyond those discussed here. For a comprehensive comparison, Table 3 summarizes the reviewed emulators alongside other notable implementations, evaluating metrics such as transistor count, maximum operating frequency, power consumption, and circuit configuration (floating/grounded). This comparative analysis provides a comprehensive perspective on the trade-offs among complexity, functionality, and practical applicability in memristor emulation. Among the reviewed implementations, DTMOS-based designs demonstrate superior high-frequency performance, with [20] achieving 5 GHz operation at a 65 nm technology node while maintaining zero static power consumption. In contrast, CMOS-based implementations show greater variability, with power consumption ranging from nanowatts to milliwatts and operating frequencies ranging from kilohertz to gigahertz, which are heavily influenced by transistor counts and passive component integration. The grounded versus floating configuration indicates that floating topologies are more common in high-performance designs, likely due to their bidirectional operation capabilities, while grounded configurations may offer implementation simplicity at the cost of flexibility. The inclusion of passive components, particularly capacitors, appears more prevalent in CMOS designs, suggesting their utility in achieving desired memristive characteristics.

### 3.2. Active Analog Emulators

While MOS-only and passive memristor emulators offer compact and low-power solutions, active analog emulator circuits provide enhanced programmability, better signal conditioning, and improved emulation accuracy through the use of operational amplifiers (op-amps), current conveyors, and other active components. These designs leverage analog circuit techniques to more precisely mimic the nonlinear dynamics and memory effects of ideal memristors, making them particularly valuable for research prototyping, neuromorphic computing, and signal processing applications. Unlike their passive counterparts, active emulators often incorporate tunable resistances, adaptive biasing, and feedback mechanisms to achieve controllable memristance modulation, enabling more accurate replication of memristive hysteresis under varying input conditions. However, this increased functionality comes at the cost of higher power consumption and circuit complexity, requiring careful trade-offs between precision and efficiency. Recent advancements in this category have focused on CMOS-compatible designs that balance programmability with integration feasibility, paving the way for hybrid analog–digital memristive systems.

To systematically analyze these developments, we classify the published works into four major subcategories based on their core active elements and structural characteristics:

- Current-mode active block-based emulators: Utilizing components such as second-generation current conveyor (CCII), current conveying transconductance amplifier (CCTA), current backward transconductance amplifier (CBTA), current follower transconductance amplifier (CFTA), current controlled current differencing transconductance amplifier (CCCDTA), these circuits leverage current-mode signal processing for high-frequency operation and compact designs.
- OTA-based emulators: Employing operational transconductance amplifiers (OTAs), these designs offer tunable memristance but often face trade-offs between frequency response and power consumption.
- Voltage differencing-based emulators: Built around voltage differencing transconductance amplifiers (VDTAs), voltage differencing current conveyors (VDCCs), and transconductance amplifiers (DVCCTAs), these circuits provide enhanced flexibility and high-frequency performance, making them suitable for reconfigurable applications.
- Mixed active element-based emulators: Combining multiple building blocks (e.g., CCIIs, OTAs, multipliers), these topologies enable complex memristive behaviors but at the cost of increased circuit complexity.

#### 3.2.1. Current-Mode Active Block-Based Emulators

The field of CCTA-based memristor emulation has demonstrated significant technological progression, marked by iterative improvements in both functional capabilities and operational performance. The designs introduced by Ranjan et al. demonstrated the feasibility of CCTA-based emulation, achieving dual-mode operation through innovative exploitation of the active component’s transconductance characteristics [55]. These preliminary circuits operated effectively in the KHz frequency range, though they exhibited limitations in bandwidth and lacked comprehensive reliability verification. The absence of advanced robustness analyses and restricted operational frequencies represented notable constraints in these initial implementations. Subsequent developments by Ranjan et al. substantially improved upon these foundations by extending the operational bandwidth to 5 MHz while maintaining the dual-mode functionality [56]. These enhanced designs incorporated rigorous verification methodologies, including non-ideal analysis and Monte Carlo simulations, thereby establishing more reliable performance benchmarks. However, these improvements came with increased circuit complexity (38 CMOS transistors) and persistent limitations in tunability. The memristor emulator proposed in [57] represents a significant advancement in CCTA-based designs by integrating electronic tunability with robust MHz-range operation. The design leverages a CCTA to achieve both floating and grounded configurations, supporting incremental/decremental modes—a flexibility critical for adaptive circuits. Unlike the CCTA emulators proposed in [55,56], this work introduces bias-current tunability, enabling dynamic control of memristance without structural modifications. Implemented in 0.18 μm CMOS technology, the emulator operates at ±1 V, demonstrating improved power efficiency over prior high-voltage designs while maintaining 20 MHz as the maximum operating frequency range. Through Cadence Virtuoso simulations, the authors conduct exhaustive analyses: short-term non-volatility tests, process corner evaluations, temperature variation, and Monte Carlo simulations. Practical validation was achieved through experimental implementation with off-the-shelf components, confirming the design’s feasibility for real-world applications.

**Figure 4:**
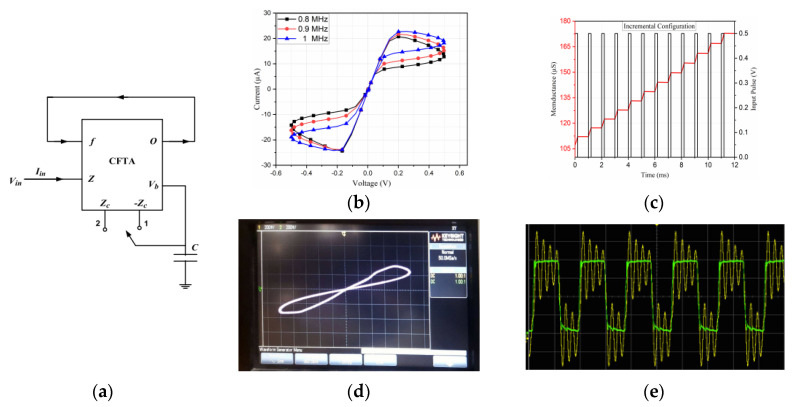
Resistorless memristor emulator using CFTA: (**a**) proposed memristor emulator circuit, (**b**) simulated pinched hysteresis loop, (**c**) non-volatile nature of memristor, (**d**) experimental pinched hysteresis loop, and (**e**) experimental Chua’s oscillator [62].

In contrast to these CCTA-based approaches, recent work [62] has demonstrated the potential for simplified architectures through a compact, grounded CFTA-based implementation. Utilizing only 28 transistors in conjunction with a grounded capacitor, this design achieves comparable MHz-range performance up to 9 MHz while reducing component count. As depicted in Figure 4, the comprehensive validation approach, incorporating both PSPICE simulations using 0.18 µm CMOS technology and experimental breadboard implementation, along with robustness assessments through Monte Carlo and non-volatility analyses, provides compelling evidence of practical viability. The successful application in Chua’s chaotic circuit further underscores the design’s functional capabilities (Figure 4e). Future work could focus on improving power efficiency, scalability, and thermal stability for broader adoption in memristive applications.

**Figure 5:**
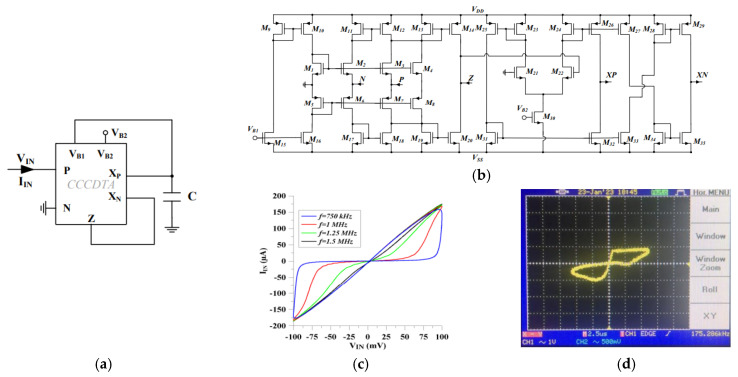
Flux-controlled memristor emulator based on a CCCDTA: (**a**) proposed memristor emulator circuit, (**b**) CMOS implementation of the CCCDTA circuit, (**c**) simulated pinched hysteresis loop, and (**d**) experimental pinched hysteresis loop at 175 KHz [59].

The authors in [58] present a memristor emulator model using a CCCCTA implemented using 32 MOSFET transistors, two resistors, and one capacitor. Operating up to 1 MHz with a compact chip area, the emulator supports both incremental and decremental configurations via a simple switch. Simulations in 180 nm CMOS technology and ±1.1 V power supply confirm theoretical predictions, demonstrating moderate power consumption of 2.2 mW and robustness through Monte Carlo, non-ideal, post-layout, and noise analyses. Experimental validation with off-the-shelf ICs further verifies the design. Also, the emulator’s applicability is showcased in chaos generation and emulating amoeba’s adaptive behavior. Furthermore, the authors in [59] introduce a flux-controlled memristor emulator based also on a CCCDTA and a grounded capacitor. The CCCDTA circuit has been designed using 35 MOSFET transistors, as depicted in Figure 5. The design features tunable invariant and variant parts, operates up to 1.5 MHz, and eliminates the need for external circuitry or topology modifications to switch between incremental and decremental modes. Performance validation via Monte Carlo and temperature analyses in TSMC 0.18 µm CMOS technology and ±0.9 V power supply confirms robust operation. The emulator consumes a low power consumption of 715 µW. Experimental verification using off-the-shelf components (ALD1116, AD844, LM13700) further supports its practicality. Comparative studies highlight the emulator’s superior performance in key metrics, reinforcing its viability for modern analog applications.

The emulator circuit designs proposed in [15,16] utilize second-generation current conveyors. The design in [15] employs a current-controlled CCII with a single grounded capacitor, demonstrating hard-switching behavior without requiring multipliers, biasing sources, or suffering from body effects. Fabricated in TSMC 0.18 μm technology, it occupies a compact 81.85 × 87.76 μm area and shows robust performance through Monte Carlo simulations across various operating conditions. The design introduced in [16] combines a CCII with a PMOS circuit to create voltage-controlled memductance, achieving 40 MHz operation frequency with only 10 transistors, which represents around 58% reduction compared to conventional CCII designs, while consuming 2.6 mW power. Implemented in 180 nm CMOS, this current-mode approach was experimentally validated using CD-4007 ICs and demonstrates read–write capability. Both designs offer significant advantages for IC implementation, including minimal component count, process scalability, and compatibility with standard CMOS fabrication, making them particularly suitable for analog memory and neuromorphic applications.

The discussed designs demonstrate remarkable improvements in transistor count reduction, operational frequency, and power efficiency, while maintaining robustness against process variations, temperature fluctuations, and noise. Where earlier studies focused primarily on functional verification, modern designs incorporate Monte Carlo analysis, temperature sweeps, and post-layout simulations. Several implementations that now include experimental validation using off-the-shelf components further confirm their practical viability.

#### 3.2.2. OTA-Based Emulators

Memristor emulation using OTAs has been extensively explored due to their tunability, compact design, and compatibility with CMOS technology. Several studies have proposed different configurations of OTA-based memristor emulators, each offering unique advantages in terms of circuit simplicity, frequency range, and application potential. The investigation in [49] presents a grounded, incremental memristor emulator based on six OTAs along with resistors and a capacitor, designed to mimic the behavior of a titanium dioxide (TiO_2_) memristor. The circuit is implemented using commercially available ICs, making it practical for both educational and real-world applications. While functional up to 1 KHz, its high component count and ±10 V supply limited its efficiency. Similarly, ref. [50] reduced the active components to four OTAs, implementing a floating emulator with extended frequency operation up to 5 KHz. However, it did not support incremental or decremental configuration. This design also emulates the HP TiO_2_ memristor model and is validated through simulations and experiments, demonstrating its viability for circuit applications. Another approach is seen in [13], where a charge-controlled memristor emulator is proposed, capable of operating in both incremental and decremental modes via a simple switch. The design employs four multi-output OTAs (MO-OTAs) in CMOS technology, allowing electronic tuning of the memristance through transconductance adjustment. The circuit maintains its pinched hysteresis loop up to 500 KHz by varying the capacitor value with a ±2.5 V supply, making it suitable for mid-frequency applications. Experimental validation using the CA3080 OTA IC confirms functionality up to 120 KHz, and a high-pass filter application demonstrates its practical utility.

Further advancements in flexibility and simplicity are demonstrated in [51], where a grounded and floating memristor emulator with a maximum operating frequency of 8 MHz is realized using only two OTAs and a grounded capacitor. The circuit supports both incremental and decremental configurations and is successfully applied in amplitude modulation (AM). The design is verified using Cadence Virtuoso Spectre, with post-layout simulations and experimental results confirming its effectiveness. An even more compact solution is proposed in [52], utilizing an MO-OTA and a single grounded capacitor to create a floating memristor emulator. This design eliminates the need for additional multiplier circuits, simplifying the architecture while maintaining nonlinear memristive behavior. The layout, implemented in TSMC 0.18 µm technology, occupies a minimal area of 34 µm × 14 µm and exhibits robust performance across varying frequencies, process corners, and temperature conditions. While its 1 MHz frequency was lower than [51], its ultra-low area made it highly integrable. However, it lacked experimental validation. The authors in [53] introduce a flux-controlled memristor emulator using only one OTA, a multi-output transconductance amplifier, a resistor, and a capacitor. This design supports both floating and grounded configurations with incremental/decremental switching via simple connection changes. Simulated in LT-Spice with 0.18 μm CMOS technology, it operates at ±1.5 V and achieves a 20 MHz operating frequency higher than most previous works. The memristance remains electronically tunable via OTA transconductance, while capacitor adjustments help maintain pinched hysteresis at higher frequencies.

**Figure 6:**
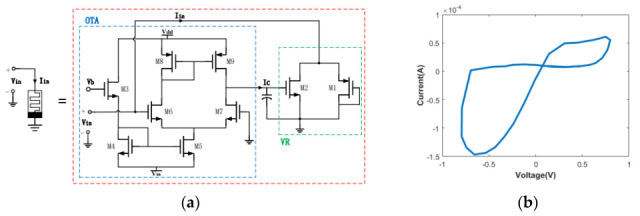
OTA-based memristor emulator: (**a**) transistor level implementation and (**b**) simulated pinched hysteresis loop at 300 MHz [14].

The authors in [14] introduce a highly efficient grounded memristor emulator utilizing a minimal component configuration consisting of a single OTA, two transistors, and one grounded capacitor, as illustrated in Figure 6. This compact design demonstrates high performance characteristics, achieving a maximum operational frequency up to 300 MHz while operating at a low supply voltage of 0.9 V. The emulator is integrated with a voltage-controlled oscillator featuring a dual-delay architecture; the complete system generates chaotic oscillations across a frequency range of 158–286 MHz. This implementation exhibits power efficiency with a peak consumption of 3.5553 mW and occupies a remarkably small silicon area of 0.0072 mm^2^ when fabricated in a 180 nm CMOS process. The chaotic nature of these oscillations has been rigorously verified through Lyapunov exponent analysis, yielding values between 0.2572 and 0.4341. These combined attributes of high-frequency operation, minimal power requirements, and compact form factor make this implementation particularly suitable for advanced applications in secure communications and neuromorphic computing systems.

This section has reviewed several OTA-based memristor emulator circuits from the literature, highlighting their distinct architectures and performance characteristics. The discussed implementations demonstrate the versatility of OTAs in designing compact, low-power, and high-frequency memristor emulators suitable for various analog and mixed-signal applications.

#### 3.2.3. Voltage Differencing Based Emulators

**Figure 7:**
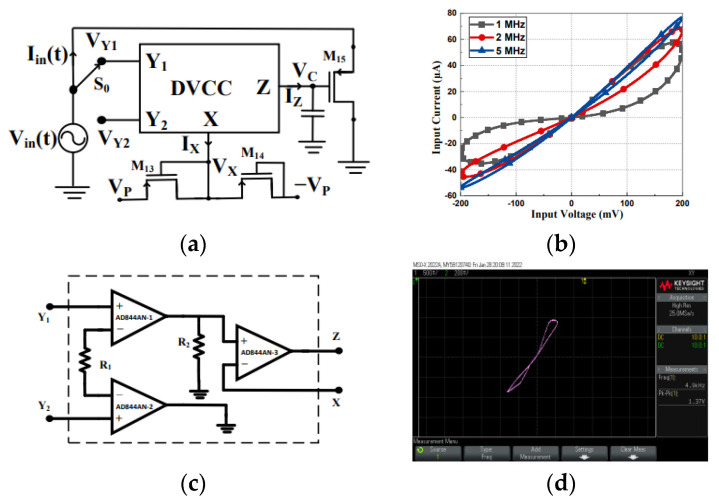
Memristor emulator based on a VDCC. (**a**) Proposed memristor emulator circuit, (**b**) simulated pinched hysteresis loop, (**c**) DVCC implementation using AD844AN, and (**d**) experimental pinched hysteresis loop at 5 KHz [69].

Memristor emulation circuits based on voltage differencing techniques have gained significant attention due to their compact design, electronic tunability, and compatibility with CMOS technology. Several researchers have proposed different architectures using voltage-differencing active blocks to achieve floating and grounded memristor emulators with incremental and decremental operation modes. A compact memristor emulator using a single DVCCTA was introduced in [63], which eliminates the need for additional components like adders and multipliers. The design operates at ±1 V with a power consumption of 8.74 mW and exhibits a wide voltage range (50–500 mV) while maintaining operation up to 12.8 MHz. The emulator was validated through non-ideal, Monte-Carlo, and temperature variation analyses, and its practical implementation was demonstrated using AD844AN and CA3080 ICs. Similarly, ref. [117] presented a DVCCTA-based emulator using a 0.25 µm CMOS process, capable of operating up to 1 MHz. The design was tested for non-volatility and implemented in a high-pass filter configuration to compare its behavior with traditional resistors. Another approach using VDTAs was proposed in [64,65], where a fully floating memelement emulator was designed with only two VDTAs and two grounded passive components. This configuration supports both memristor and meminductor behaviors without requiring external multipliers, offering electronic tunability for neural network applications. The design was verified using PSPICE simulations and commercial IC-based implementations. In [66], fully balanced voltage differencing buffered amplifiers (FB-VDBA) were employed to develop simple, efficient memristor emulators. These designs maintain pinched hysteresis loops up to 1 MHz and can easily switch between incremental and decremental modes. The floating configuration was further utilized in a universal biquad filter to assess its performance. A similar study in [67] introduced electronically tunable memristor emulators using a VDGA, maintaining hysteresis characteristics from 5 KHz to 1 MHz. These emulators were applied in analog filter designs to verify their functionality. A simpler approach was adopted in [68], where a VDCC was used with only two PMOS transistors and a grounded capacitor. The design allows electronic tuning of memductance and operating frequency, with experimental validation using discrete components. Additionally, the authors in [69] have proposed a 100 MHz flux-controlled memristor emulator using a single DVCC, one capacitor, and three PMOS transistors, where two form an active resistor and one serves as a multiplier. The design supports reconfigurable operation in incremental/decremental modes via a simple switch and exhibits non-volatile behavior by retaining states without external stimuli, as depicted in Figure 7. Validated in 180 nm CMOS technology and experimentally verified using AD844AN and CD4007 ICs, the emulator demonstrates robustness against process variations through PVT and Monte Carlo analyses. As a practical application, a neuromorphic adaptive learning circuit is implemented, showcasing its potential for brain-inspired computing systems.

Further advancements include voltage differencing inverting buffered amplifier (VDIBA)-based designs, as seen in [70], where a resistorless, tunable memristor emulator was proposed. The circuit operates up to 12.7 MHz with a low power consumption of 1.34 mW and was validated through extensive robustness tests. Similarly, Yeşil et al. [71] and Vista and Ranjan [72] introduced voltage difference transconductance amplifier (VDTA)-based emulators with a single active element and grounded capacitor, supporting both incremental and decremental modes. These designs were experimentally verified using commercial ICs (LM13700 and CA3080) and applied in neuromorphic learning circuits. In summary, voltage-differencing-based memristor emulators offer advantages such as compactness, electronic tunability, and high-frequency operation. Various active blocks, including DVCCTA, VDTA, FB-VDBA, VDCC, and VDTA, have been successfully employed, with experimental validations confirming their suitability for practical applications in various domains.

#### 3.2.4. Mixed Active Element-Based Emulators

Memristor emulators utilizing mixed active components have been widely explored to achieve enhanced functionality, electronic tunability, and compact circuit design. These emulators combine different active building blocks such as current backward transconductance amplifiers (CBTAs), CCIIs, OTAs, VDCCs, DVCCs, and multipliers to realize both grounded and floating configurations with incremental and decremental operation modes. A CBTA-based memristor emulator was proposed in [77], employing only one CBTA, two resistors, one capacitor, and a multiplier. The design allows switching between incremental and decremental modes by simply interchanging CBTA output terminals. Implemented in 0.18 µm CMOS technology, the circuit operates at ±0.9 V and demonstrates a wide output range with a moderate transistor count (23 CMOS transistors). Similarly, ref. [78] introduced a voltage-differencing transconductance amplifier (VDTA)-based floating memristor emulator, requiring only one VDTA, two resistors, one capacitor, and a multiplier. The design supports operation up to 2 MHz and was experimentally validated using commercial ICs. Several studies have explored CCII-based emulators associated with other active blocs. For example, the authors in [79] presented a current-controlled memristor emulator using two CCIIs (AD844 ICs), a multiplier (AD633), and an AOP (TL082). Another approach in [80] combined a CCII with an OTA to realize a flux-controlled memristor emulator operating up to 26.3 MHz. The design was verified through non-volatility tests, Monte Carlo analysis, and experimental breadboard implementation using AD844AN and CA3080 ICs. In [81], a current-controlled CCTA was used along with an OTA to design resistorless grounded and floating memristor emulators. This emulator was tested in a chaotic Jerk circuit, demonstrating its applicability in nonlinear systems. Another study [82] combined an OTA with a current differencing buffered amplifier (CDBA) to develop simple yet efficient memristor emulators maintaining pinched hysteresis loops up to 1 MHz. In [83], a fully floating memristor emulator was designed using a VDCC and an OTA-controlled resistor, replicating silicon nanowire (SiNW) memristive behavior. The emulator demonstrated non-volatile characteristics and was validated through simulations and experiments. Similarly, ref. [84] introduced a VDCC-OTA-based charge-controlled memelement emulator capable of realizing both memristor and memcapacitor behaviors without external multipliers. Advanced hybrid configurations have also been explored. For example, ref. [85] proposed a voltage differential transconductance amplifier (VDTA) and voltage differential complementary amplifier (VDCA)-based floating memristor emulator with long-term memory (LTM) capabilities. The design prevents charge leakage and operates stably at 10 MHz. Another hybrid approach in [86] utilized a dual-output OTA (DO-OTA) and DVCC to develop a fully floating emulator with electronically adjustable memductance. High-frequency operation has been a key focus in recent designs. Ref. [73] introduced a current-mode-based memristor emulator operating up to 30 MHz, using two active blocks and two passive components. The emulator was implemented in 180 nm CMOS technology and tested in neuromorphic adaptive learning circuits. Similarly, ref. [87] proposed a resistorless, electronically tunable memristor emulator using OTA and VDBA, maintaining hysteresis loops up to 5 MHz and demonstrating applications in binary frequency shift keying (BFSK).

#### 3.2.5. Comparative Analysis of Analog Memristor Emulators: Performance Metrics and Design Trade-Offs

Table 4 presents a comparative analysis of analog active memristor emulators based on several key performance metrics. The table systematically organizes published works by their reference number, year of publication, and critical design parameters, including the number of active and passive components, operation modes (incremental/decremental), configuration type (grounded/floating), transistor count, power supply requirements, power consumption, maximum operating frequency, tunability features, and experimental validation status.

Recent advancements in memristor emulator circuits demonstrate a clear shift to simpler, more efficient, and high-performance designs. Early implementations relied on multiple active components (e.g., CCIIs, OTAs, multipliers) and several passive elements, resulting in complex circuits with limited operating frequencies (often below 1 MHz). However, modern emulators leverage single active blocks such as DVCCTA, VDTA, FB-VDBA, and DVCC, significantly reducing transistor count while improving frequency response. For instance, ref. [63] achieves 12.8 MHz operation with just one DVCCTA, while the recent work published in 2024 introduced in [74] pushes the limit to 500 MHz, the highest reported frequency, using a similar approach. Another notable new research interest is the move toward resistorless designs, where only a single capacitor is used [66,68,87], enhancing IC compatibility and reducing parasitic effects. Tunability and reconfigurability have also become key features in recent emulators. Many designs now support electronic control of memristance via bias voltages or transconductance adjustments [63,74,78,88], allowing dynamic switching between incremental and decremental modes without circuit modifications. Additionally, floating configurations are increasingly favored for their various applications, such as analog and neuromorphic applications. Power efficiency has significantly improved, with advanced emulators such as in [69] consuming only 7.64 µW at 100 MHz, significantly lower than earlier designs that required ±10 V power supplies and had much higher power consumption. Experimental validation remains crucial, with many works demonstrating real-world feasibility using off-the-shelf ICs (AD844AN, CA3080, LT1193) [63,68,69,70,80]. However, some high-frequency designs [71,74] still rely on simulations, indicating a need for further hardware verification. Overall, the progression from bulky, low-frequency emulators to ultra-compact, high-speed, and tunable circuits highlights the field’s maturation, paving the way for integration into neuromorphic systems, adaptive filters, and high-frequency signal processing. Future efforts may focus on improving non-volatility, reducing power further, and enhancing CMOS compatibility for broader adoption.

### 3.3. Nonlinear Passive Emulators

Memristor emulation using nonlinear circuit elements has garnered significant attention as a practical approach to replicate the unique properties of memristors without requiring nanoscale fabrication. Early designs leveraged the voltage-dependent resistance characteristics of diode–resistor networks to mimic memristive behavior, offering a simple yet effective solution for experimental research and circuit applications. While these emulators successfully demonstrated key memristor features, such as pinched hysteresis loops, their performance was often constrained by bandwidth limitations and discrete resistance states. This section reviews the evolution of such emulator circuits, highlighting their working principles, design improvements, and inherent challenges, particularly in achieving continuous state transitions and higher operating frequencies.

Early research explored the nonlinear behavior of diode–resistor networks for memristor emulation [79,93,94,95,96,97,98]. These designs rely on the principle that a diode’s threshold voltage alters the circuit’s effective resistance. In 2011, Biolek and Bajer introduced the first grounded emulators based on this concept [93,94]. This study presents an analog method for emulating a memristor using its defined charge-flux constitutive relationship. The emulation is achieved through a nonlinear resistor, characterized by its current–voltage behavior, combined with a mutator circuit. The mutator performs a similarity transformation, converting the resistor’s characteristic into the memristor’s constitutive. The nonlinear resistor was constructed using an LED (LA-541B) and two resistors, exhibiting a piecewise-linear characteristic with a breakpoint near 2 V. The test results demonstrate accurate memristor emulation. However, the op-amp’s limited gain-bandwidth product restricts its operational frequency to a few hundred hertz. Later work optimized this approach by replacing light-emitting diodes with germanium variants for lower turn-on voltage and substituting op-amps with current-feedback amplifiers (CFOAs) to mitigate bandwidth constraints [79]. While this improved frequency performance slightly, it remained below the kilohertz range. Authors in [95] have developed a floating emulator operating at kilohertz frequencies, though its symmetry requirement for component values posed a challenge: mismatches disrupted current balance, hindering accurate floating memristor simulation. This issue was addressed through tunable capacitors and resistors.

Simpler implementations, such as diode bridges paired with passive components (resistors, capacitors, inductors), also replicate memristive behavior [97,98] and find use in chaos circuits. However, these designs cannot achieve continuous resistance states, only discrete steps, even with added components. Despite this limitation, their straightforward architecture makes them suitable for logic gates or applications requiring binary resistance states. Additionally, several works propose voltage-controlled memristor emulators using diode-based circuits. The authors in [99] introduce a generalized memristor consisting of a memristive diode bridge with a parallel RC filter, demonstrating pinched hysteresis loops under different stimuli and validating its operation up to 10 KHz. Similarly, ref. [100] presents an autonomous floating memristive circuit using only a second-order memristive diode bridge and a capacitor. This design exhibits complex dynamics, including unipolar periodic oscillations, chaotic bursting, and coexisting attractors, a behavior not previously observed in third-order autonomous memristive systems. The study also employs spectral entropy complexity for parameter optimization, with experimental verification confirming theoretical predictions. Expanding beyond single-phase designs, ref. [101] investigates memristive characteristics in a three-phase diode bridge rectifier. The analysis reveals pinched hysteresis loops with additional intersection points in the first and third quadrants under normal operation, while fault conditions further alter the hysteresis behavior. The circuit operates at 20 KHz and is classified as a generalized memristor.

**Figure 8:**
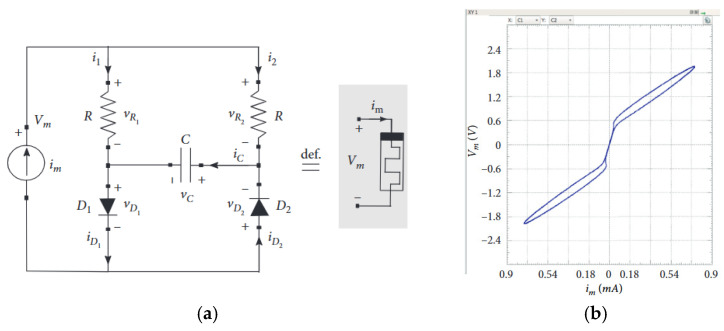
(**a**) Current-controlled memristor circuit proposed in [102] and (**b**) its simulated pinched hysteresis loop.

Meanwhile, ref. [102] addresses a critical gap in memristor emulation by proposing a passive current-controlled inductorless emulator using only two diodes, two resistors, and a capacitor, as depicted in Figure 8. Unlike previous designs, which were predominantly voltage-controlled, this circuit enables current-driven memristive behavior, validated through simulations up to 3 KHz and experiments up to 1.5 KHz. Higher-frequency applications are explored in [103], which introduces a third-order autonomous chaotic circuit with a current-controlled memristor, achieving an operating frequency of 150 MHz. The system exhibits abrupt transitions from periodic limit cycles to chaos, differing from conventional smooth bifurcation routes. In contrast, ref. [104] focuses on a second-order passive current-controlled memristor using minimal components (one capacitor, one inductor, two resistors, and two diodes). This design enables hyperchaotic behavior, with numerical and circuit simulations confirming periodic, quasiperiodic, chaotic, and hyperchaotic attractors at frequencies up to 50 KHz.

Table 5 summarizes key characteristics of memristor emulator circuits reported in the literature, comparing their implementation complexity (active/passive components), topology (grounded/floating), operating frequency, and experimental validation status to identify design performance trade-offs. The reviewed studies demonstrate significant progress in memristor emulation, showcasing diverse circuit topologies that replicate memristive behavior using passive and active components. Voltage-controlled designs, such as diode-based RC filters and three-phase rectifiers, provide reliable emulation with pinched hysteresis loops, while novel current-controlled approaches overcome previous limitations, enabling inductorless and passive implementations.

### 3.4. Digital Emulators

The development of digital memristor emulators has undergone significant evolution over the past decade, progressing from simple microcontroller-based implementations [109,110,111,112,113,114] to sophisticated FPGA [105,106,107,108] architectures. This evolution has been driven by the need for higher precision, faster response times, and greater flexibility in memristor emulation for various applications, ranging from neuromorphic computing to programmable analog circuits. This section reviews key developments in digital memristor emulation, analyzing their architectures, performance, and suitability for different applications.

The first generation of digital memristor emulators emerged in the early 2010s, primarily utilizing microcontroller-digital potentiometer (DigPot) architectures [109]. These designs were motivated by the lack of commercially available memristors and the need for a programmable alternative. The authors have introduced a microcontroller-based emulator where the resistance of a DigPot was dynamically adjusted in real-time based on voltage measurements from an analog-to-digital converter (ADC). This approach allowed the emulator to replicate voltage-controlled memristive behavior, enabling applications such as tunable threshold circuits and frequency modulators. However, this early design was limited by the microcontroller’s processing speed and the DigPot’s resolution, resulting in non-ideal memristive hysteresis at higher frequencies. Building on this concept, the authors in [110] have simplified the architecture using an Arduino microcontroller and a serial peripheral interface (SPI)-controlled DigPot, making the emulator more accessible for educational and prototyping purposes. The Arduino sampled the voltage across the DigPot, computed the target resistance using memristor equations, and updated the DigPot accordingly. While this implementation was cost-effective and easy to replicate, it inherited the same limitations as [109], including ADC noise and latency issues. Nevertheless, these early microcontroller-based emulators demonstrated the feasibility of digital memristor emulation and laid the groundwork for more advanced designs. Recent years have seen refinements in microcontroller-based emulators, particularly in improving their programmability and reducing analog noise. For example, Strukov et al. (2008) [2] introduced an optoelectronic variant that replaced the traditional DigPot with a hand-made optocoupler, allowing for light-controlled resistance tuning. This design was applied to low-pass (LP) and high-pass (HP) filters with adjustable cutoff frequencies, showcasing improved tunability compared to purely electronic DigPot-based emulators. However, optocouplers introduced their own nonlinearities, and the overall system still suffered from the microcontroller’s bandwidth constraints. A recent example published in 2023 includes a microcontroller-based emulator using an STM32F103RB [111]. The study presents a low-cost, hand-made optocoupler-integrated emulator applied to an adjustable LP filter, demonstrating practical utility in tunable frequency response. Experimental validation confirms stable performance, though the design’s scalability and high-frequency operation remain unaddressed. Although improvements were made over proposed emulators, microcontroller-based emulators remained fundamentally limited by their sequential processing, making them unsuitable for high-speed or parallel computing tasks. Researchers turned to FPGAs for high-speed, parallelizable memristor emulation. FPGAs offered several advantages, including reconfigurable logic, low-latency digital signal processing, and the ability to implement complex mathematical models in hardware.

**Figure 9:**
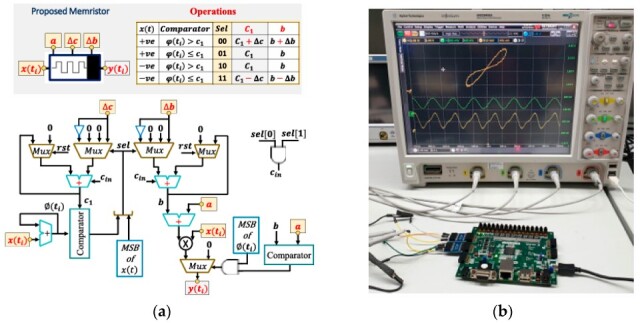
(**a**) Proposed memristor model hardware architecture, and (**b**) experimental results for proposed memristor model I–V characteristic [105].

The authors in [105] have presented an FPGA-based emulator, which implemented a versatile digital memristor model capable of mimicking both continuous and discrete memristive behaviors, such as those seen in electrochemical metallization memory (ECM) and valence change memory (VCM). Figure 9a presents the hardware architecture of the proposed memristor model, including the top module and the 2-bit selector, which are designed based on constant operations and the datapath circuit. Figure 9b shows the experimental setup and the resulting I–V characteristics of the proposed memristor emulator. This emulator was used to design a chaotic oscillator for speech encryption, demonstrating FPGAs’ capability to handle nonlinear dynamics in real time. The design achieved high throughput with minimal resource utilization on an Xilinx Artix-7 FPGA, paving the way for more complex applications. Further advancements in FPGA emulation focused on fractional-order memristor models, which better capture the memory and nonlinear characteristics of nanoscale memristive devices. The authors in [18] introduced an FPGA architecture based on the Grünwald–Letnikov approximation, enabling efficient computation of fractional-order calculus with reduced memory and computational overhead. This approach allowed for real-time emulation of fractional-order memristors with adjustable parameters, making it suitable for advanced signal processing and control systems. The most recent developments in FPGA-based emulation also target neuromorphic computing and edge AI applications.

The study in [19] presents an FPGA-based memristor emulator circuit designed for efficient binary convolution operations, utilizing a multi-bit XNOR gate as its primary computational block, paired with a memristor-inspired pooling layer. The binary convolutional neural network (BCNN) layer is implemented using a cascaded XNOR-NOR logic structure, optimized for hardware efficiency. The design was successfully synthesized and tested on an Xilinx Nexys4 FPGA, demonstrating minimal resource usage (<1% utilization) and high-speed operation at 144.9 MHz. Experimental validation confirmed that the hardware implementation achieves performance comparable to software simulations, verifying the functional accuracy of the proposed emulator architecture. Another work introduces a digital emulator of a voltage-controlled bipolar memristor, implemented on low-cost FPGAs to replicate synaptic properties [106]. Validated in an artificial neural network as an associative memory, this emulator demonstrates the feasibility of using digital solutions for neuromorphic systems where commercial memristors remain expensive. The study in [107] proposes a general discrete memristor emulator with an innovative frequency-shifting (GDME) technique to overcome noise and bandwidth limitations in pinched hysteresis loop measurements. By processing signals digitally on an FPGA-based platform, the emulator achieves an experimental maximum operating frequency of 20 GHz—3300 times better than existing methods. Additionally, the work explores coupled GDME configurations for stochastic computing, showcasing improved dynamic performance in chaotic systems. Additionally, the authors in [108] present a multivalued memristor model emulating both continuous and discrete behaviors, similar to electrochemical metallization memories. Synthesized on an Xilinx ZYNQ-7000 FPGA with <1% hardware utilization, the emulator is tested in an eight-valued quantized ANN, achieving 92.8% accuracy on the MNIST dataset. This highlights its potential for efficient, high-precision neural network implementations.

As discussed, recent implementations, including microcontroller-based, Raspberry Pi-driven, and FPGA-accelerated designs, demonstrate the adaptability of digital emulators across various applications from tunable filters to neuromorphic computing and real-time image processing. While microcontroller solutions provide cost-effective prototyping, FPGA-based emulators excel in high-speed, parallel operations, and software-programmable approaches enable dynamic reconfigurability. However, challenges remain in achieving true analog memristor behavior, minimizing power consumption, and ensuring robustness under real-world conditions.

### 3.5. Hybrid Emulators

Recent research has shown growing interest in developing hybrid memristor emulators, with a focus on enhancing their flexibility, power efficiency, and scalability for use in neural networks, signal processing, and edge computing. The authors in [115] introduce an FPGA-based gate-controlled memristor emulator, designed to overcome the limitations of traditional two-terminal memristors in large-scale neural networks. By leveraging the local linearity of device conductance, the design efficiently implements a multiply-accumulate circuit, achieving less than 1% hardware utilization on an Xilinx XQ7Z020 FPGA. When tested on a four-state quantized single-layer perceptron using the MNIST dataset, it achieved 87.6% recognition accuracy, closely matching software simulations with only a 0.03% deviation. This approach highlights the potential of FPGA-based emulators in energy-efficient neural network acceleration. Another similar study presents a Raspberry Pi-based digital memristor emulator, addressing reconfigurability issues in analog designs [116]. By tuning gate voltage parameters, the emulator mimics different memristor behaviors while operating at 500 MHz. In a motion detection application, it processed a 640 × 350-pixel video stream with 53 mW power consumption and a 3.52 µs delay, demonstrating its suitability for real-time, low-power edge computing tasks. The authors in [17] propose a mixed-signal switched-capacitor memristor emulator, combining analog and digital techniques for cost-effective memristance control. Using an FPGA to generate precise switching signals, the design emulates memristive behavior with high accuracy. This approach is particularly useful for applications requiring low-cost, reconfigurable analog computing, such as adaptive filters or neuromorphic systems. These studies illustrate the advantages of hybrid emulators. However, challenges remain in improving analog fidelity, reducing power consumption, and scaling up for large networks. Future research should explore hybrid analog–digital architectures to combine the strengths of these approaches while addressing their limitations.

## 4. Applications of Memristor Emulators in Healthcare Systems

Memristor emulator circuits serve as essential tools for investigating memristive characteristics and their integration into real-world electronic systems. Analyzing these emulators in practical circuits not only facilitates the transition toward commercial memristor applications but also provides valuable insights into their functional relevance. Extensive research demonstrates the deployment of memristor emulators across diverse circuit configurations, including filters [12,80,89], logic circuits [12,38], digital modulation systems [38,42,51,55,79,95], Schmitt triggers [42,109,110], and oscillators [12,89]. While memristor emulators have shown great promise in general electronics, their potential in healthcare is particularly exciting. In the next section, we explore how these versatile circuits are being adapted for medical applications, including synapse design, physiological signal processing, and medical image processing. Their unique ability to mimic biological synapses and process signals efficiently makes them ideal for advancing healthcare technologies.

**Figure 10:**
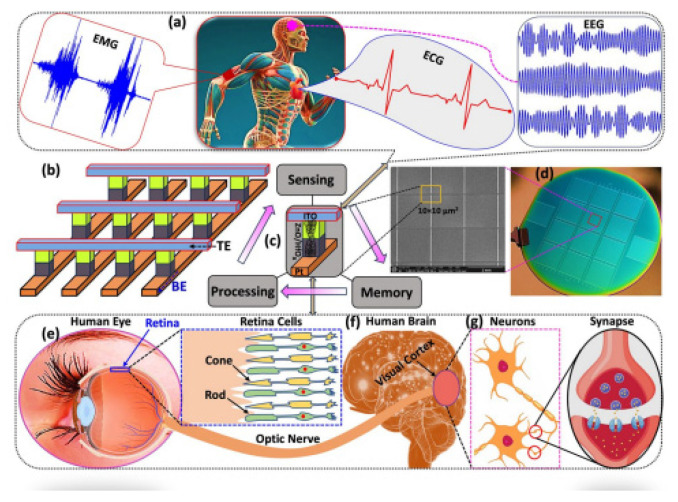
Schematic diagram of the physiological signal processing framework: (**a**) Physiological signals (EEG, EMG, and ECG), (**b**) Crossbar array architecture, (**c**) Sensing-memory-processing operation of the device, (**d**) Optical photograph of the crossbar array, (**e**) Detailed structure of the human retina, (**f**) Diagram of the human brain, and (**g**) Schematic of neurotransmitter activity between pre-synaptic and post-synaptic sites in the retina [118].

Memristor-based circuits are emerging as a transformative technology for physiological signal processing, offering unprecedented advantages in real-time biosignal analysis, adaptive filtering, and low-power medical diagnostics. Figure 10 presents the schematic diagram of a physiological signal processing framework that captures data from key organs, including the brain (EEG), muscles (EMG), and heart (ECG). Non-invasive EEG signals enable the reconstruction of neural activity patterns and eye movement detection for identity verification, while EMG assesses neuromuscular function to diagnose disorders. Simultaneously, ECG recordings monitor cardiac electrical activity, aiding in the detection of arrhythmias and heart conditions. This integrated approach aligns with a crossbar array architecture, which facilitates efficient signal processing and data storage. The device combines sensing, memory, and computing functions, mimicking biological systems, such as the human visual pathway, comprising the retina, optic nerve, and brain, where synaptic-like neurotransmitter interactions occur between neurons. Notably, memristors offer a promising opportunity for enhancing this system, as their ability to emulate synaptic plasticity and perform in-memory computing can improve energy efficiency and processing speed. Their integration could enable advanced neuromorphic designs, bridging the gap between biological signal processing and artificial intelligence. Furthermore, their compact design and in-memory computing capabilities make them ideal for wearable health monitors, implantable devices, and edge-computing medical IoT systems, paving the way for smarter, more responsive healthcare technologies.

**Figure 11:**
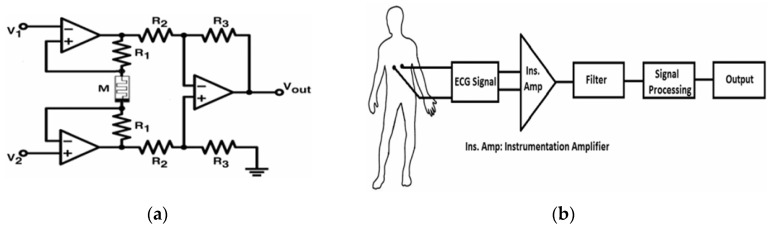
(**a**) Instrumentation amplifier using memristor and (**b**) ECG measurement system using memristor-based instrumentation amplifier [119].

Figure 11 presents a block diagram of an ECG measurement system introduced in [119], employing an instrumentation amplifier (IA) enhanced with a memristor as a programmable gain element. Instrumentation amplifiers are critical in biomedical signal acquisition due to their ability to amplify weak physiological signals (in the millivolt-to-microvolt range) while rejecting noise and interference. The three-amplifier IA configuration leverages the memristor’s tunable resistance to dynamically adjust the gain, replacing traditional fixed resistors. All passive components in the circuit use 10 kΩ resistors, while the memristor enables adaptive gain control tailored to specific signal-processing needs. For validation, a 1 KHz input signal of 2 mV peak-to-peak was applied, yielding an amplified output of 0.84 V in differential mode and a gain of 420. In common-mode operation, the output measured just 20.98 mV, corresponding to a minimal common-mode gain of 0.42. This performance translates to a common-mode rejection ratio of 60 dB, demonstrating the system’s ability to robustly amplify bioelectric signals while suppressing noise. The memristor’s integration thus enhances the IA’s versatility, enabling real-time gain adjustment for optimal ECG signal fidelity in dynamic environments.

**Figure 12:**
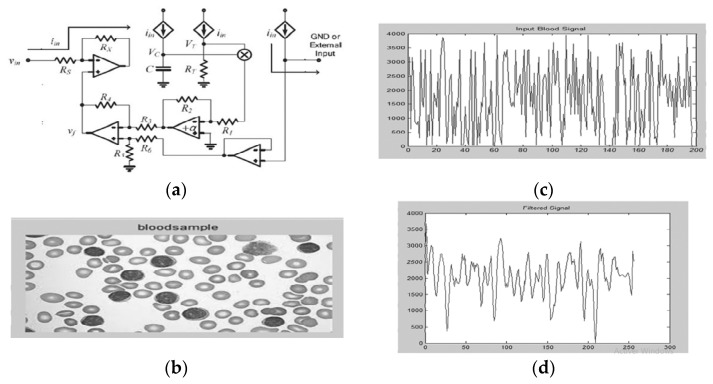
(**a**) Memristor emulator circuit diagram, (**b**) input blood sample, (**c**) input signal for 15-SG FIR filter, and (**d**) filtered output of blood signal [120].

The authors in [120] demonstrate their application through a 15-tap continuous-time FIR Savitzky–Golay filter design using memristor-based delay blocks, specifically optimized for processing slow-varying blood signals. The proposed memristor emulator circuit to implement the storage element is depicted in Figure 12. The simulation results using MATLAB and ModelSim verify the approach’s effectiveness for handling physiological waveforms while maintaining the signal fidelity, a crucial advantage over digital quantization methods for medical applications. The demonstrated memristor emulator architecture bridges theoretical research with practical medical device needs, particularly for wearable health monitoring systems requiring low-power, analog processing of biological signals. This work expands upon previous neuromorphic computing applications by providing a concrete implementation example for biosignal filtering, complementing existing pattern classification approaches while addressing the distinct challenge of continuous physiological waveform processing in resource-constrained edge devices.

The analysis of individual physiological signals often faces limitations in diagnostic accuracy, prompting researchers to explore multi-signal fusion approaches. Combining complementary biosignals like ECG and PPG improves robustness in cardiovascular monitoring, but traditional fusion methods suffer from high power consumption and processing latency. Memristor-based circuits address these challenges by enabling parallel, energy-efficient processing of multiple time-series signals through their inherent memory properties and analog computing capabilities. This technology is particularly suited for wearable devices where power constraints and real-time processing are critical, offering a hardware solution to overcome current fusion analysis bottlenecks. Spiking neural networks (SNNs) have emerged as a biological framework for processing physiological time-series data like EEG and ECG, as they naturally encode temporal spike patterns [121]. Memristors serve as ideal hardware synapses for SNN implementation, with their conductance modulation perfectly mimicking synaptic plasticity. This approach allows event-driven processing that matches the discrete nature of biosignals, native temporal pattern recognition without complex analog-to-digital conversion, and ultra-low power operation through in-memory computing. For example, the SRAM-based approach presented in [122] achieves remarkable digital processing efficiency (51.4 TOPS/W) through innovative multicycle addition, making it suitable for high-speed operations but limited by volatility and area constraints. In contrast, the phase-change memory solution introduced in [123] leverages analog multilevel storage capability, though with higher energy costs and limited endurance compared to emerging alternatives. The RRAM implementation in [124] stands out with exceptional energy efficiency around 1.2 POps/s/W and compact analog processing, validating resistive memory’s potential for neural network acceleration.

Recent demonstrations of memristor-based SNNs for ECG classification highlight their potential to revolutionize edge-computing medical devices by combining the efficiency of neuromorphic architectures with the adaptive signal fusion capabilities of memristive circuits. As an example, the authors in [125] present a full CMOS-compatible, circuit-level implementation of an SNN system featuring on-chip training and classification using memristive STDP synapses in standard 180 nm technology. This hardware innovation demonstrates particular promise for medical applications, as evidenced by a successful adaptation of the system for heart rate classification. By modifying the STDP circuit to achieve Bienenstock–Cooper–Munro (BCM) characteristics, they have created a versatile platform for processing various physiological signals. The architecture’s advantages, including elimination of external processors (FPGAs/CPUs/GPUs), reduced system area, and lower power consumption, directly address the challenges of multi-signal fusion discussed earlier. This approach significantly outperforms previous implementations in both energy efficiency and processing speed, making it ideally suited for wearable health monitors that require the real-time analysis of combined ECG, PPG, and other biosignals while maintaining stringent power budgets.

Modern diagnostic medicine increasingly relies on multimodal medical imaging, where complementary techniques like CT and MRI provide synergistic clinical information. However, these imaging modalities face inherent challenges, including noise artifacts that compromise diagnostic accuracy and the need for precise edge detection in anatomical structures. To address these limitations, the authors in [126] have introduced a novel memristive pulse-coupled neural network (M-PCNN) architecture that leverages the unique properties of Gale-type memristors for enhanced medical image processing. The exponential time-decay characteristic of memristance enables dynamic threshold adjustment in our PCNN implementation, mimicking biological neural adaptation while maintaining compact hardware requirements. This bio-inspired approach demonstrates three key clinical applications: cross-modal image fusion combining CT and MRI data for improved tumor localization in radiation therapy planning, adaptive noise suppression preserving critical anatomical features, and high-precision edge detection for surgical planning. Simulation results confirm the network’s superior performance in these medical imaging tasks, while the nanoscale memristor integration enables future miniaturized hardware implementations, a critical advancement for point-of-care diagnostic systems. This work establishes a framework for implementing neuromorphic image processing in clinical environments, potentially transforming workflows in neuroradiology and surgical oncology. Furthermore, the authors in [127] present a novel memristor-based CNN architecture. The system directly converts pixel values into corresponding voltage pulses, which are processed through memristor crossbar arrays serving as both memory and computational elements. When these input pulses exceed a predefined threshold voltage, the output neurons generate responses, mimicking the efficient processing of biological visual systems. This innovative approach eliminates the traditional von Neumann bottleneck by performing computations directly within the memory structure, enabling unprecedented processing speeds while maintaining ultra-low power consumption.

The growing body of research highlights the significant potential of memristors in medical and healthcare applications, including physiological signal processing [128], humanoid neural computing [129], digital image enhancement [130], and neuromorphic sensing [131]. Memristive systems offer superior energy efficiency, faster processing, and bio-inspired dynamics compared to traditional von Neumann architectures, making them ideal for real-time diagnostics, robotic medicine, and advanced medical imaging. However, while numerous theoretical and simulation-based studies demonstrate their capabilities, practical implementation remains limited. To bridge this gap, prototyping memristor-based systems using emulators is a crucial next step, enabling experimental validation and accelerating their adoption in clinical and biomedical environments. Future efforts should focus on scalable fabrication, integration with existing medical hardware, and overcoming device variability to fully realize the transformative impact of memristive technologies in healthcare.

Furthermore, the optimization of memristor emulators for medical applications fundamentally depends on the specific clinical use case. For biosignal acquisition and processing in monitoring devices, the design must prioritize high sensitivity to microvolt-level signals while maintaining exceptional noise rejection capabilities. This is achieved through precision analog front-end circuits with adaptive biasing and programmable filtering characteristics. In contrast, diagnostic imaging applications, such as MRI and CT reconstruction, require architectures optimized for parallel computation and fast switching speeds, where the emphasis shifts toward high throughput and moderate precision operations. Key engineering trade-offs emerge when adapting these emulators for healthcare environments. Wearable health monitors demand ultra-low-power operation (<10 μW) with robust motion artifact rejection, while neural interfaces require high temporal resolution for spike detection and chronic stability in biological environments. The most significant compromise exists between signal fidelity and power consumption, where implantable devices demand ultra-low-power operation at the expense of some precision, while diagnostic equipment can accommodate higher power budgets for improved accuracy. Similarly, speed requirements for real-time processing must be balanced against the physical constraints of wearable or implantable form factors. These competing demands necessitate flexible architectures that can be customized for specific clinical applications while maintaining core functionality.

## 5. Challenges and Future Directions

Memristor emulator circuits have emerged as a practical alternative to physical memristors, enabling researchers to explore memristive behavior without the challenges of nanoscale fabrication. However, several technical and practical limitations must be addressed to advance their adoption in real-world applications. This section outlines the key challenges and suggests future research directions to overcome them.

Despite the significant progress in memristor emulator circuits, several challenges still limit their widespread adoption and practical implementation. A fundamental challenge in memristor emulator development lies in achieving accurate replication of real memristive device behavior. Current emulator designs predominantly rely on simplified mathematical models, such as linear ion drift or nonlinear threshold switching approximations, or they implement circuit topologies that superficially mimic memristive behavior without a proper mathematical foundation or empirical validation. However, these approaches fail to capture several critical non-ideal characteristics observed in physical memristive devices. The stochastic nature of memristor operation presents a significant modeling hurdle. Physical devices exhibit cycle-to-cycle variability due to the probabilistic nature of conductive filament formation and rupture in oxide-based memristors. For example, TiO_2_-based devices demonstrate substantial resistance fluctuations between switching cycles, with variations often exceeding 20% of nominal values. In contrast, most emulator circuits assume deterministic and perfectly repeatable switching behavior, leading to discrepancies between emulated and actual device performance. Another critical limitation is the inability of current emulators to account for device degradation over time. Practical memristive devices experience gradual performance deterioration with cycling, as seen in TaOₓ-based systems, which typically show measurable degradation after approximately 10^6^ switching cycles. This aging effect, which significantly impacts long-term reliability, remains largely unaddressed in existing emulator implementations. Furthermore, the frequency-dependent nature of memristor hysteresis poses additional challenges. While most emulators implement static hysteresis models, actual devices such as HfO_2_-based memristors demonstrate strongly frequency-dependent pinched hysteresis loops. This dynamic behavior becomes particularly problematic when emulating high-speed switching applications or complex neuromorphic circuits where timing-dependent effects are crucial for proper operation. Also, nonlinearities in the current–voltage characteristics further complicate their modeling, as asymmetrical responses and threshold drifts make it difficult to achieve consistent and reliable emulation.

Furthermore, the inherent variability and non-ideality of emulator components present a limitation of published emulators. Since most memristor emulators rely on active circuits such as operational amplifiers, multipliers, or floating passive elements, their performance is highly sensitive to component tolerances, temperature fluctuations, and aging effects. These factors introduce deviations from the expected pinched hysteresis behavior, leading to inaccuracies in mimicking true memristive dynamics. Power consumption and scalability present another major challenge. Emulator circuits often require complex active components, leading to high static power dissipation that is impractical for energy-efficient neuromorphic systems. Some designs demand large bias voltages or currents, conflicting with modern low-voltage CMOS technology and limiting their compatibility with standard integrated circuits. Moreover, area inefficiency is a critical concern. Many emulator topologies use multiple transistors or bulky passive elements, occupying excessive chip space and limiting their use in large-scale crossbar arrays or high-density computing architectures. Emulating the non-volatile nature of physical memristors remains an unsolved challenge for most circuit implementations. Analog emulators typically lose their state when power is removed, while digital approaches require continuous power to maintain memory contents. Proposed solutions, such as floating-gate transistors or integrated non-volatile memory, add substantial complexity to the circuit design. Furthermore, charge leakage in analog storage elements and refresh requirements in digital implementations introduce additional power overhead and reliability concerns. Achieving true non-volatility without compromising other performance metrics remains an active area of research. Additionally, the field currently lacks standardized methodologies for evaluating and comparing memristor emulator performance. Different research groups use varying metrics and test conditions, making it difficult to assess the relative merits of different approaches. There is no consensus on critical parameters such as acceptable hysteresis error, minimum operational frequency range, or power efficiency benchmarks. This lack of standardization hinders progress in the field and complicates the transition from academic research to commercial applications. Also, the absence of comprehensive reliability testing protocols makes it challenging to evaluate the long-term stability and robustness of emulator circuits under real operating conditions. To address the current lack of consistent benchmarking in memristor emulator research, we propose the following standardized evaluation framework. First, mathematical accuracy should be quantified through hysteresis loop error measurements, for example, <5% deviation for precision applications, and <10% for basic implementations, against established models like VTEAM or nonlinear drift. Second, frequency response characterization can adopt a three-tier classification system: low-frequency operation (DC–1 KHz), medium-frequency range (1 KHz–1 MHz), and high-frequency capability (>1 MHz) for advanced computing, analog signal processing, and high-speed memory applications. Third, power efficiency metrics should include both static consumption and dynamic switching energy, as these critically impact scalability. Fourth, reliability testing should assess cycle-to-cycle variability and endurance. Finally, all evaluations should use standardized test conditions, including defined input waveforms, load impedances, and environmental controls to ensure reproducible comparisons. This framework not only enables fair performance assessment but also discourages superficial implementations by requiring mathematical rigor and empirical validation. By adopting these criteria, researchers can better evaluate emulator suitability for specific applications like neuromorphic, memory, and analog computing, while facilitating meaningful cross-study comparisons and technology transfer.

These challenges collectively represent significant barriers that must be overcome to realize the full potential of memristor emulator technology. While each issue presents its own set of complexities, they are interrelated; improvements in one area often require trade-offs in another. Addressing these limitations requires a multi-disciplinary approach that combines novel circuit architectures, advanced materials, and intelligent system integration. Future research should focus on developing solutions that not only overcome current constraints but also expand the potential applications of memristor emulation technology. Several promising directions emerge as particularly critical for advancing the field.

Future emulator designs must break through current frequency limitations to enable applications in RF and high-speed signal processing. Research should explore current-mode architectures using advanced active blocks like CFOAs or CCIIs, which offer higher bandwidth characteristics. Nanoscale CMOS implementations in advanced process nodes below 45 nm could significantly reduce parasitic effects while improving switching speeds. Hybrid analog-digital approaches that combine the speed of analog processing with the precision of digital control may provide the best path toward GHz-range operation while maintaining memristive characteristics. Emerging technologies such as silicon photonics could also be investigated for optical memristor emulation, potentially offering wide bandwidth capabilities. Additionally, the development of ultra-low-power emulator circuits is essential for wireless and healthcare applications. Future work should investigate subthreshold CMOS designs that operate at supply voltages below 0.5 V, significantly reducing dynamic power consumption. Novel architectures could incorporate energy harvesting techniques to create self-powered emulator systems, particularly for IoT and biomedical applications. Memristor-emulator-based compute-in-memory architectures should be optimized to minimize energy-per-operation metrics, potentially approaching the efficiency of physical memristor devices. Research into passive and semi-passive emulator topologies that minimize active component count while maintaining functionality could yield substantial power savings for large-scale implementations. Another promising direction involves achieving reliable non-volatility in emulator circuits requires innovative approaches to state retention. Future research should explore the integration of emerging non-volatile memory technologies, such as ferroelectric FETs or magnetoresistive elements, with conventional emulator circuits. Advanced floating-gate designs using ultra-thin oxide layers could provide improved charge retention characteristics. Digital emulators could incorporate novel non-volatile flip-flop designs or emerging resistive RAM cells for state storage. Hybrid solutions that combine the speed of volatile emulation with periodic non-volatile backups may offer a practical compromise for certain applications. The development of standardized retention metrics and testing protocols will be crucial for evaluating these approaches. Furthermore, future research should move beyond generic emulator designs and focus on application-optimized implementations tailored to specific domains. For example, neuromorphic computing involves developing emulators with biologically realistic synaptic plasticity rules and enhanced STDP characteristics. In biomedical applications, the priority should be on creating ultra-low-power, biocompatible emulators for implantable neural interfaces and biosignal processing. For edge AI systems, researchers should design compact emulator arrays optimized for in-memory computing and machine learning acceleration. Meanwhile, RF systems would benefit from high-frequency emulators with adaptive impedance matching capabilities. By following these specialized directions, emulator technology can better address the unique demands of each field. To advance memristor emulation technology, future research should focus on its integration with emerging computing and novel material platforms. Promising directions include developing quantum-inspired architectures to bridge neuromorphic and quantum computing systems, as well as cryogenic-compatible designs for superconducting electronics applications. Tunable implementations could enhance wearable healthcare and bioelectronic systems’ performances, while photonic approaches may overcome speed and energy limitations in conventional designs.

Furthermore, to address common non-idealities in memristor emulator circuits, several practical design strategies can be implemented. To address parasitic effects, such as stray capacitance and series resistance, compensation techniques that actively counteract these non-idealities can be used. For example, dummy elements or negative impedance converters can be employed to neutralize undesired impedance, ensuring that the emulator’s behavior remains faithful to ideal memristive models. This approach is particularly critical in high-frequency applications where parasitic components significantly distort dynamic responses. Beyond compensation, maintaining linearity across varying operating conditions is another key challenge. Many emulator circuits suffer from nonlinearity due to transistor mismatches or voltage/current limitations. To mitigate this, adaptive biasing techniques, such as dynamically adjusting gate voltages in MOSFET-based emulators or using programmable current mirrors, can be explored. These methods help preserve linearity over a wide range of input signals, making the emulator more versatile for practical applications. Stability under environmental fluctuations is also important, which is where feedback-based control proves invaluable. By integrating feedback loops, either through operational amplifiers or digital control systems, the emulator can self-correct in real time, minimizing deviations caused by temperature drift or supply noise. This not only enhances accuracy but also ensures consistent hysteresis behavior, a defining feature of memristive systems. For large-scale implementations, modular and scalable architectures offer a practical solution. Discrete-time emulation techniques, such as sample-and-hold circuits or FPGA-based designs, allow for precise tuning and easy replication, making them suitable for integrated systems where uniformity is essential. These approaches bridge the gap between theoretical models and real-world deployable circuits. On the calibration front, automated parameter tuning plays a pivotal role in optimizing performance. Algorithms can dynamically adjust resistance thresholds and other key parameters, compensating for component tolerances. This ensures that the emulator adheres closely to its intended behavior, even when individual parts exhibit minor variations. For long-term reliability, real-time adaptive calibration further refines the emulator’s accuracy. For example, embedded analog-to-digital converters can continuously monitor the circuit’s state, enabling closed-loop adjustments that correct drift over time. This is especially useful in applications where sustained operation is critical, such as neuromorphic computing or adaptive filtering.

These research directions represent a roadmap for overcoming current limitations while expanding the capabilities of memristor emulator technology. Successful development in these areas could enable transformative applications across computing, communications, and biomedical engineering, bridging the gap between emulated and physical memristive systems. The interdisciplinary nature of these challenges will require close collaboration between circuit designers, materials scientists, and application domain experts to achieve significant progress.

## 6. Conclusions

Memristor emulator circuits have emerged as a versatile and cost-effective alternative to physical memristors, enabling advancements in various fields. This review paper has comprehensively examined various memristor emulator circuits, analyzing their working principles, design methodologies, and key characteristics. We have highlighted their significant potential in healthcare applications, including biosignal processing and medical image processing. By summarizing the latest advancements and critical challenges, such as stability, scalability, and integration issues, this work provides a valuable reference for researchers developing reliable and efficient memristor-based systems. Future progress in this field will depend on refining emulator architectures while strategically incorporating emerging technological innovations to enhance electronic systems.

## Figures and Tables

**Table 1:** Comparative analysis of memristor models.

Model	Device Type	State Variable	Control Variable	Captures Threshold	Frequency Range	Main Advantage	Limitation
Chua Model	Generic	Charge/Flux linkage	Current	No	<10 KHz	Fundamental, general theory	Abstract, not device-specific
HP Model (linear Drift)	Bipolar	0 ≤ w ≤ D (Doped region Width)	Current	No	10 KHz	Simple, physically intuitive	Ignores nonlinearities, boundary effects
Nonlinear Drift	Bipolar	0 ≤ w ≤ 1 (Normalized width)	voltage	No	100 KHz	More accurate than linear drift	Empirical window function, not universal
Simmons tunneling barrier	Bipolar	a_off_ ≤ w ≤ a_on_ (Barrier width)	Current	No	1 MHz	Quantum-mechanically rigorous for tunneling	Computationally intensive, less general
Yakopcic	Bipolar	0 ≤ w ≤ 1 (Empirical)	Voltage	Yes	10 MHz	Highly accurate, fits many devices	Requires extensive parameter extraction
TEAM	Bipolar	x_on_ ≤ x ≤ x_off_ (Threshold-based)	Current	Yes	50 MHz	Flexible, efficient for digital circuits	Needs tuning for different devices
VTEAM	Bipolar	x_on_ ≤ x ≤ x_off_ (Threshold-based)	Voltage	Yes	100 MHz	Voltage-controlled, better for analog/ReRAM	Complex parameter calibration

**Table 3:** Comparative analysis of CMOS-based memristor emulators.

Ref	Year	Type	MOSFET Count	Passive Component Count	Grounded/Floating	Technology Used	Power Consumption	Frequency	Experiment Completed
[11]	2024	DTMOS	5	0	Both	0.18 µm	0	500 MHz	Yes
[12]	2025	DTMOS	4	0	Floating	0.18 µm	0	250 MHz	No
[13]	2023	CMOS-based	2, 1 CS	0	Floating	180 µm	4 μW	1 GHz	No
[20]	2023	DTMOS	4	0	Both	65 nm	0	5 GHz	Yes
[21]	2019	CMOS-based	4	0	Grounded	0.18 µm	40 µW	100 KHz	Yes
[22]	2020	DTMOS	3	0	Both	0.18 µm	0	20/30 MHz	Yes
[23]	2023	CMOS-based	2	0	Grounded	65 nm	963 µW	300 MHz	Yes
[24]	2023	DTMOS	4	1	Floating	180 µm	8.24 µW	3 MHz	Yes
[25]	2024	DTMOS	1	R-1, C-1	Both	45 nm	7.75 pW	80 MHz	Yes
[37]	2023	CMOS-based	3	1	Grounded	90 nm	175 nW (dynamic)	24 MHz	Yes
[38]	2022	CMOS-based	4	0	Floating	90 nm	2.6 µW	50 MHz	Yes
[39]	2018	CMOS-based	4	0	Grounded	0.18 µm	128.7 µW	100 MHz	Yes
[40]	2025	CMOS-based	2	C-1	Both	65 nm	47 µW	150 MHz	No
[41]	2025	CMOS-based	6	C-1	Grounded	0.18 µm	NA	NA	No
[42]	2019	CMOS-based	3, 1 CS	C-1	Floating	0.18 µm	6.725 nW	13 MHz	Yes
[43]	2017	CMOS-based	3	C-1	Grounded	0.18 µm	0	100 KHz	No
[44]	2018	CMOS-based	7	C-1	Grounded	0.18 µm	NA	50 MHz	Yes
[45]	2017	CMOS-based	6	C-1	Floating	0.18 µm	NA	10 Hz	No
[46]	2018	CMOS-based	7	0	Floating	0.13 µm	NA	1 MHz	No
[47]	2023	CMOS-based	9	C-1	Grounded	45 nm	NA	2 KHz	No
[48]	2022	CMOS-based	8	C-1	Grounded	180 µm	46 mW	100 MHz	Yes

**Table 4:** Comparative analysis of active analog memristor emulators.

Ref	Year	No. of Active Components	No. of Passive Components	Inc/Dec	G/F	No. of MOS	Power Supply	Power Consumption	Max Operating Frequency	Tunability	Experiment Complete
[90]	2013	2 CCII, 1 multiplier	4 R, 3 C	Both	G	-	-	-	270 KHz	No	Yes
[79]	2014	2 CFOA, 1 OTA	3 R, 2 C	Both	G	42	-	-	Few KHz	No	Yes
[89]	2014	4 CCII, 1 multiplier 1 Op-Amp	10 R, 1 C		F	-	-		Few Hz	No	Yes
[49]	2015	6 OTA	2 R, 1 C	Inc.	G	-	±10 V	-	1 KHz	Yes	Yes
[50]	2015	4 OTA	4 R, 1 C	-	F	-	±10 V	-	5 KHz	-	Yes
[55]	2017	1 CCTA	3 R, 1 C	Both	Both	30	±1.5 V	7.5 mW	10 MHz	Yes	Yes
[117]	2017	1 DVCCTA	3 R, 1 C	Both	G	29	±1.25 V	-	1 MHz	No	Yes
[77]	2017	1 CBTA, 1 multiplier	2 R, 1 C	Both	G	23	±0.9 V	-	460 KHz	Yes	No
[91]	2017	1 CCII, 1 multiplier	1 C, 1R	Both	G	-	±10 V	-	860 KHz	No	Yes
[54]	2017	2 transistors 1 OTA	1C, 0R	Both	Both	16	±1 V	-	30 Hz	No	No
[92]	2017	1 MO-OTA, 1 multiplier	1C, 1R	Both	G	>38	±1.25 V	-	1 KHz	Yes	Yes
[13]	2018	4 MO-OTA	1 C, 3 R	Both	G	92	±2.5 V	-	150 KHz	No	Yes
[51]	2018	2 OTA	1 C, 0 R	Both	Both	34	±1.2 V	-	8 MHz	Yes	Yes
[52]	2018	1 MO-OTA	1 C, 0 R	Both	Both	17	±0.9 V	-	1 MHz	No	No
[71]	2018	1 VDTA	1 C	Both	G	16	±0.9 V	-	50 MHz	No	Yes
[78]	2018	1 VDTA, 1 multiplier	1 C, 2 R	Both	F	32	±0.9 V	-	2 MHz	Yes	Yes
[56]	2019	1 CCTA, 1 CCII	3 R, 1 C	Both	G	38	±1.5 V	-	5 MHz	No	Yes
[68]	2019	1 VDCC, 2 CMOS	1 C	Both	G	26	±0.9 V	-	2 MHz	Yes	Yes
[60]	2019	1 CCCDTA	1 C	Both	G	35	±2.5 V	-	1 MHz	No	No
[72]	2020	1 VDTA	1 R, 1 C	Both	Both	16	±0.9 V	8 µW	50 MHz	Yes	Yes
[80]	2020	1 CCII, 1 OTA	1 C, 1 R	Both	G	13	±1.2 V	9.567 mW	26.3 MHz	No	No
[82]	2020	1 CDBA, 1 OTA	1 C	Both	Both	27	±0.9 V	-	1 MHz	Yes	No
[86]	2020	1 DVCC, 1 DO-OTA, 2 Mosfets	1 C	Inc.	F	29	±1.2 V	-	1.5 MHz	Yes	Yes
[88]	2020	1 CDTA 1 OTA	1 C, 0 R	Both	Both	36	±0.9 V	-	2 MHz	Yes	No
[15]	2021	1 CCCII	1 C	Inc.	G	9	±0.9 V	-	7.5 MHz	No	No
[62]	2021	1 CFTA	1 C	Both	G	28	±0.9 V	-	9 MHz	No	Yes
[64]	2021	2 VDTA	1 R, 1 C	Inc.	F	32	±0.9 V	-	1.5 MHz	Yes	No
[65]	2021	2 MVDCC	1 C, 2 R	Inc.	F	52	±0.9 V	-	500 KHz	Yes	Yes
[66]	2021	1 FB-VDBA	1 C, 0 R	Both	Both	19	±0.9 V		1 MHz	No	No
[67]	2021	1 VDGA	1 C	Both	F	33	±0.8 V	-	1 MHz	Yes	No
[84]	2021	1 VDCC, 1 OTA	2 R, 1 C	Inc.	G	35	-	-	1 MHz	Yes	Yes
[61]	2021	1 CDTA, 4 MOS	1 C	Both	F	24	±1.2 V	-	100 MHz	No	No
[53]	2022	1 MO-OTA, 1 OTA	1 R, 1 C	Both	Both	42	±1.5 V	-	20 MHz	No	No
[63]	2022	1 DVCCTA	2 R, 1 C	Both	Both	27	±1 V	8.74 m	12.8 M	Yes	Yes
[70]	2022	2 VDIBA	1 C	Both	F	-	±1 V	1.34 mW	12.7 MHz	Yes	Yes
[76]	2022	2 VDCC, 2 MOSFETs	1 C	Both	Both	46	±0.9 V	-	50 MHz	Yes	No
[16]	2023	1 CCII, 1 MOSFET	1 R, 1 C	Inc.	G	10	±1.5 V	2.6 mW	40 MHz	No	Yes
[59]	2023	1 CCCDTA	1 C	**Inc.**	G	35	±0.9 V	715 µW	1.5 MHz	Yes	Yes
[69]	2023	1 DVCC, 3 MOS	1C	Both	G	15	±1.25 V	7.64 µW	100 MHz	Yes	Yes
[73]	2023	1 DVCC, 1 OTA	1 R, 1 C	Both	G	23	±0.9 V	591 µW	30 MHz	Yes	Yes
[87]	2023	1 OTA, 1 VDBA	1 MOS-Cap	Both	Both	25	±0.9 V	-	5 MHz	Yes	Yes
[75]	2023	1 MVDTA	1 R, 2 C	-	Both	50	±0.9 V	1 mW	500 KHz	Yes	Yes
[57]	2024	1 CCTA	2 R, 1 C	Both	Both	30	±3 V	18 mW	20 MHz	Yes	Yes
[81]	2024	1 CCCCTA, 1 OTA	1 C	Dec.	Both	34	±0.9 V	7.2 μW	15 MHz	Yes	No
[83]	2024	1 OTA, 1 VDCC	1 R, 1 C	Both	F	-	±3.3 V	-	10 KHz	Yes	Yes
[74]	2024	1 DVCCTA	2 R	Both	Both	-	±0.9 V	4.84 mW	500 MHz	Yes	No
[14]	2025	1 OTA, 2 Mosefts	2 R, 1 C	**Inc.**	G	9	±0.9 V	-	300 MHz	Yes	No
[58]	2025	1 CCCCTA	2 R, 1 C	**Dec.**	Both	32	±1.1 V	2.2 mW	1 MHz	Yes	Yes
[85]	2025	1 VDTA-VDCA	1 C, 0 R	Both	Both	21	±5 V	-	10 MHz	Yes	No

**Table 5:** Comparative analysis of nonlinear passive emulators.

Ref	Year	No. of Active Components	No. of Passive Components	Grounded/Floating	Max Operating Frequency	Experiment Complete
[93]	2011	1 LED, 4 TOA	2R, 2C	Grounded	100 Hz	Yes
[98]	2012	4 Diodes	3 (1C, 1L, 1R)	Grounded	1 KHz	No
[99]	2014	4 Diodes	2 (1C, 1R)	Grounded	10 KHz	No
[79]	2014	1 Diode, 3 CFOA	3R, 2C	Grounded	700 Hz	Yes
[96]	2016	2 BJTs, 2 Diodes	6 (2C, 4R)	Floating	10 KHz	Yes
[100]	2019	4 Diodes	2 (1C, 1L)	Floating	10 KHz	Yes
[101]	2019	6 Diodes	2 (1C, 1R)	Grounded	20 KHz	No
[102]	2021	2 Diodes	3 (1C, 2R)	Grounded	3 KHz	Yes
[103]	2021	2 Diodes	3 (1C, 2R)	Grounded	150 KHz	No
[104]	2022	2 Diodes	4 (1C, 1L, 2R)	Grounded	50 KHz	No

## Data Availability

Data are contained within the article.
